# Genomic Uniqueness of Local Sheep Breeds From Morocco

**DOI:** 10.3389/fgene.2021.723599

**Published:** 2021-12-02

**Authors:** Abdessamad Ouhrouch, Simon Boitard, Frédéric Boyer, Bertrand Servin, Anne Da Silva, François Pompanon, Abdelmajid Haddioui, Badr Benjelloun

**Affiliations:** ^1^ Livestock Genomics Laboratory, Regional Center of Agricultural Research Tadla, National Institute of Agricultural Research INRA, Rabat, Morocco; ^2^ Biotechnologies and Valorization of Plant-Genetic Resources Laboratory, Sultan Moulay Slimane University, Beni Mellal, Morocco; ^3^ CBGP, Université de Montpellier, CIRAD, INRAE, Institut Agro, IRD, Montpellier, France; ^4^ Université Grenoble Alpes, Université Savoie MT-Blanc, CNRS, LECA, Grenoble, France; ^5^ GenPhySE, Université de Toulouse, INRA, INPT, INP-ENVT, Castanet-Tolosan, France; ^6^ PEREINE/E2LIM, Faculty of Science and Technics, Limoges, France

**Keywords:** sheep, whole genome sequences, local breeds, demography, selection signatures

## Abstract

Sheep farming is a major source of meat in Morocco and plays a key role in the country’s agriculture. This study aims at characterizing the whole-genome diversity and demographic history of the main Moroccan sheep breeds, as well as to identify selection signatures within and between breeds. Whole genome data from 87 individuals representing the five predominant local breeds were used to estimate their level of neutral genetic diversity and to infer the variation of their effective population size over time. In addition, we used two methods to detect selection signatures: either for detecting selective sweeps within each breed separately or by detecting differentially selected regions by contrasting different breeds. We identified hundreds of genomic regions putatively under selection, which related to several biological terms involved in local adaptation or the expression of zootechnical performances such as Growth, UV protection, Cell maturation or Feeding behavior. The results of this study revealed selection signatures in genes that have an important role in traits of interest and increased our understanding of how genetic diversity is distributed in these local breeds. Thus, Moroccan local sheep breeds exhibit both a high genetic diversity and a large set of adaptive variations, and therefore, represent a valuable genetic resource for the conservation of sheep in the context of climate change.

## 1 Introduction

Sheep were among the first domesticated animals about 11,000 years B.P (Vigne, 2011; Demirci et al., 2013). They are one of the main sources of meat and milk around the world (Ritchie and Roser, 2017). According to the FAO, the worldwide stocks of sheep reached 1,238 M heads in 2019 with proportions of 42.6% in Asia, 32.9% in Africa, 10.3% in Europe, 7.5% in Oceania and 6.7% in the Americas (FAOSTAT, 2020). The world production increase of sheep products was about 13.7% for meat and about 9.9% for milk between 2009 and 2019 (FAOSTAT, 2020). In the context of environmental changes, the improvement and conservation of this species are major challenges to sustainably meet the growing needs of human populations for meat and dairy products, both at the national and international levels (Bruford et al., 2015). Furthermore, in order to achieve the United Nations SDG#2 (Sustainable Development Goals), the target #2.5 is directly concerned by maintaining genetic diversity in domestic species, and two associated indicators are related to the proportion of endangered breeds (#2.5.1 & #2.5.2). Thus, local traditionally-managed sheep breeds represent an officially recognized valuable genetic resource for the conservation of this species on a global scale.

Technological advances during the last decade have made it possible to produce and process Whole Genome data for hundreds of individuals (Kulski, 2016). Similarly, bioinformatic improvement has incredibly advanced (Mangul et al., 2019). The DNA sequence of an individual is the most comprehensive collection of its genetic variation, and today’s sequencing technology that is much more increasingly efficient, faster and cheaper than ever (Kulski, 2016) allows access to this genome-wide variation for many individuals from the same population or breed. This allows a precise characterization of genetic resources, by characterizing properly their demographic dynamics and geographic structuration, as well as their adaptive diversity (Benjelloun et al., 2019).

In Morocco, sheep are marked by a high global genetic diversity indicating a high adaptive potential (Benjelloun, 2015; Benjelloun et al., 2021). The main local breeds currently farmed have significant ability to adapt to their breeding system and environment. However, the genetic diversity within each breed and the demographic and adaptive history that shaped this diversity are not yet very well known. Only a few hypotheses have been emitted to describe their origin (Boujenane, 1999).

Sheep farming in Morocco plays an important economic and sociological role. It is practiced all over the country where it is often one of the main sources of farmers’ income. Thus, sheep are bred under various environmental conditions and anthropogenic pressures. One of the main threats to these breeds is the unsupervised crossing practiced by farmers under increasing economic pressure as recently demonstrated by Belabdi et al. (2019). These practices, evaluated by the FAO to establish risk status of breeds, have led to genetic dilution (FAO, 2007), to which is added the risk of replacement of local breeds by cosmopolitan breeds (Taberlet et al., 2008).

The Moroccan sheep population is made up of about 1% of exogenous breeds and 99% of local breeds (Boujenane, 2006), among which the most important ones are Sardi, Dman, Timahdite, Beni Guil and Boujaad. Since 1980, five main local sheep breeds have been standardized and officially recognized by a large management program named the National Sheep Plan (MARA, 1980) which was based on assigning theses breeds to their exclusive specific habitat or area named “cradle of the breed”. Thus, Beni Guil is bred in the Eastern plateaus in large herds using pastoralism as main feeding sources. The Dman breed has been bred in the palm groves of the pre-Saharan regions of the South-Eastern Morocco for a long time and is mainly located in the oases. Dman is considered as the most isolated and phenotypically distinguished breed in Morocco. The Sardi breed belongs to the sheep population of the western plateaus in Morocco. It is considered as a large-body sheep and very appreciated in religious events. Timahdite is a rustic breed well adapted to mountainous areas with low-input systems. Finally, the Ouled Jellal breed, which is not considered indigenous in Morocco, was shown to be among the main breeds farmed in the Eastern region of the country together with Beni Guil since a long time (Belabdi et al., 2019), and is considered as a true breed of the steppe, well adapted to nomadism.

Many characteristics that condition the fitness of livestock are associated with the production performances. Thus, the identification of signatures of selection is a valuable approach to identify the genes and polymorphisms underlying the phenotypic variation of these traits that are subjected to both anthropogenic selection (Rochus et al., 2018) and natural selection related to e.g., climatic or ecological constraints. While previous studies using WGS have identified many genome-environment associations in Moroccan sheep (Benjelloun, 2015), the genomic bases of traits specific to local breeds are still undetermined.

In this context, this study aims at characterizing the main five Moroccan breeds by describing their genomic diversity, inferring their demographic history and identifying shared and breed-specific selective sweeps using whole genome data. The data obtained enabled us to distinguish genomic regions putatively involved in local adaptation from those more directly related to anthropogenic pressures and associated to breed-specific zootechnical performances.

## 2 Methods

### 2.1 Samples and Breeds

We sampled 87 unrelated sheep representatives of the geographic distribution of five local Moroccan breeds (Sardi, Dman, Timahdite, Beni Guil and Ouled Jellal; Supplementary Table S1) across the Northern half of Morocco (North of latitude 28°) between January 2008 and March 2012 in accordance with ethical regulations of the European Union Directive 86/609/EEC, as described in Benjelloun et al. (2021) and Benjelloun (2015). Tissue samples were taken from the distal part of the ear and then placed in alcohol for 1 day, after which they were transferred to a silica gel tube pending the extraction of DNA.

Additionally, a worldwide breed panel consisting of 20 sheep representing 20 different worldwide breeds was provided by the International Sheep Genome Consortium. The panel represents sheep from Asia, Africa Australia, America and Europe (Supplementary Table S1). Similarly, 13 wild Asiatic mouflons (*O. orientalis*) were collected either from captive or recently hunted animals, and from frozen samples available at the Iranian Department of Environment. These worldwide sheep (*n* = 20) and Asiatic mouflons (*n* = 13) were previously used in Alberto et al. (2018) and their whole genome sequence data were included here for comparisons and for increasing power when identifying selection signatures in Moroccan sheep (see section 2.3 and section 2.4 in the Methods).

### 2.2 Data Processing

As described by Benjelloun et al. (2021), Alberto et al., 2018 and Benjelloun et al. (2019), Illumina reads were aligned to the sheep reference genome (OAR v3.1, GenBank assembly GCA_000317765.1 (Jiang et al., 2014), and variant discovery was performed using three different algorithms: Samtools Mpileup (Li et al., 2009), GATK UnifiedGenotyper (McKenna et al., 2010) and Freebayes (Garrison and Marth, 2012). Variants were called using a larger dataset than that used in this study (i.e., 160 sheep). Then two successive rounds of filtering variant sites were run. Filtering stage 1 merged together calls from the three algorithms, whilst filtering out the lowest-confidence calls. A variant site passed if it was called by at least two different calling algorithms with variant phred-scaled quality>30. An alternate allele at a site passed if it was called by any one of the calling algorithms, and the genotype count >0. Filtering stage 2 used Variant Quality Score Recalibration by GATK. First, a training set was generated of the highest-confidence variant sites where 1) the site was called by all three variant callers with variant phredscaled quality >100; 2) the site was biallelic, and 3) the minor allele count was at least three, counting only samples with genotype phredscaled quality >30. The training set was used to build a Gaussian model using the tool GATK VariantRecalibrator using the following variant annotations from UnifiedGenotyper: QD, HaplotypeScore, MQRankSum, ReadPosRankSum, FS, DP, Inbreeding Coefficient.

The Gaussian model was applied to the full data set, generating a VQSLOD (log odds ratio of being a true variant). Sites were filtered out if VQSLOD < cutoff value. The cutoff value was set for each population by the following: Minimum VQSLOD = {the median value of VQSLOD for training set variants}—3 * {the median absolute deviation VQSLOD of training set variants}. Measures of the transition/transversion ratio of SNPs suggest that this chosen cutoff criterion gives the best balance between selectivity and sensitivity.

Genotypes were improved and phased by Beagle 4 (Browning and Browning, 2013), and then filtered out where the genotype probability calculated by Beagle was <0.95. The resulting dataset was constituted of 47,622,950 variants.

### 2.3 Genetic Diversity and Demography

We used vcftools (Danecek et al., 2011) to estimate the heterozygosity (Ho), inbreeding coefficient (F) using polymorphic diploid bi-allelic SNPs and nucleotide diversity (π) for all diploid SNPs without missingness. We also calculated the F_ST_ index (Weir and Cockerham, 1984) using the same program (VCFtools) for each variant and their average over the whole genome between each two of the five Moroccan breeds, and between the 87 Moroccan individuals and the 22 sheep representing 12 cosmopolitan breeds.

In order to determine the demographic history of the studied breeds, we used the approximate Bayesian computation (ABC) approach implemented in PopSizeABC (Boitard et al., 2016a) to estimate the effective population size (Ne) through time from 130 K years to the present. PopSizeABC determines how Ne changes through time, by estimating empirical summary statistics from our VCF files and matching them to the simulated summary statistics obtained from simulated genomic data. The simulations explore a large set of Ne possible values for each of 21 pre-defined time windows, together with several values of the per generation per bp recombination rate (*r*), while assuming a fixed and pre-defined per generation per site mutation rate (*μ*). In order to compare the demographic history Moroccan sheep with the main worldwide breeds/populations, we included, in the analysis, genomic data of the worldwide sheep and Asiatic mouflons. The latters represent the closest wild descents of the species from which the current domestic sheep (*O. aries*) diverged since their domestication. The worldwide breeds were subjected to the improvement of their production performances by intensive selection (Alberto et al., 2018).

We estimated the recombination rate by using a uniform prior interval from 8e^−9^ to 13e^−9^ with an approximation of **r** = 10.65e^−9^, and then empirical data for each population. The minor allele count threshold (mac) for the Allele Frequency Spectrum (AFS) and Identity By State (IBS) statistics computation was set for each population to about **5% × N** (where N is the number of samples) and the minor allele count threshold for LD statistics computation (mac_ld) was set to about **20% × N** as recommended by Boitard et al., (2016a). The number of simulated datasets was nb_rep = 10,000, the number of independent segments in each dataset was nb_seg = 50 and the size of each segment in bp was L = 2,000,000, which overall gives a total of 1 Terabp of simulated data.

### 2.4 Selection Signatures

To identify selective sweeps, we have defined an integrated framework based on two complementary approaches as proposed in Boitard et al., (2016b). The first one (freqHMM) detects selection occurring within a single population while the second (FLK & hapFLK) is for detecting selection events differentiating populations.

A- A genome scan using the freqHMM program (Boitard et al., 2012) allowed identifying independently the putative regions under selection within each of the five studied breeds. This method is based on contrasting the local and genome-wide distributions of allele frequencies using data from a single population by assuming each SNP to have a hidden state which can take three different values: 1) “3 = Selection”; attributed to SNPs that are located in a selective sweep, 2) “intermediate”, for SNPs not selected but located close to a selective sweep and 3) “1 = neutral”, for SNPs that are far from any selective sweep. It aims at identifying ancient selection signatures that arose from new variants (*hard sweeps*) as described by Boitard et al. (2012). Ancestral alleles at each marker were defined using the homozygotes of the reference goat genome (CHIR_1.0; Dong et al., 2013) for the corresponding loci, by assuming that these are the ancestral alleles that mutated in *Ovis* to produce SNPs. Those ancestral alleles were added to the input VCF files using an in-house shell script. Afterwards, five different datasets representing whole genome variants with a total of polymorphic 31,442,046 SNPs (for which ancestral alleles were identified) of each of the five breeds were extracted using vcftools (Danecek et al., 2011) and data related to each chromosome was put in a separate file for each breed. Then, we calculated the number of derived and ancestral alleles for each SNP using vcftools. Parameter K of freqHMM was calibrated for each breed by simulating data using ms (Hudson, 2002) under neutrality, accounting for the demographic history previously estimated for the breed by PopSizeABC, and running freqHMM on these neutral data, as previously described in Boitard et al., (2016b). The value of K obtained by this approach was then used to run the freqHMM analysis on the empirical data for each one of the five breeds.

B- The second approach to detect selective sweeps was a combination of the FLK (Bonhomme et al., 2010) and hapFLK (Fariello et al., 2013) methods implemented in the hapFLK (1.4 version) program (https://forge-dga.jouy.inra.fr/projects/hapflk/files). It was used to detect selection signatures by differentiating haplotypes among hierarchically structured populations, as described by Fariello et al. (2013). This has been done using all the genomic autosomes with no missingness allowed. FLK is a test for inbreeding coefficient heterogeneity that uses phylogenetically estimated relationships between populations. The allele frequencies are rescaled using a population kinship matrix which is estimated from the genomic data, measuring the amount of genetic drift expected under neutral evolution. HapFLK uses the differences in the frequencies of allele haplotypes between populations and the hierarchical structure of subpopulations.

The shared variants between the five Moroccan breeds and the 19 *Ovis Orientalis* (included as outgroup) were combined using the option “-T CombineVariants” of GATK (McKenna et al., 2010). The resulting dataset consisting of 31,721,507 polymorphic SNPs was used for FLK analysis to calculate Reynolds distances and the resulting Kinship Matrix. Then, this dataset was subdivided into several files each of which has a window of 10 Mbp with an overlap of 1 Mbp between each two successive windows. Subsequently, we launched FLK/hapFLK on all files one by one with a specified number of clusters set to K = 25. The analysis was launched with the option of keeping the Outgroup when computing FLK/hapFLK scores. We applied the approach of Storey and Tibshirani (2003), implemented in the q-value R package (Storey et al., 2015) to control the false discovery rate (FDR) based on FLK/hapFLK *p*-values.

Lists of genes that include or less than 5 kb away from the identified candidate SNPs (Downstream 5′-end and upstream 3′-end) were established and used for the Gene Ontology (GO) enrichment analyses. Similarly, outlier genomic regions were constituted of 50 kb segments surrounding outlier SNPs. GO enrichment analyses were performed using GOwinda (Kofler and Schlötterer, 2012) in order to explore the biological processes in which the identified genes under selection are involved. Bos Taurus was used as the reference species for that analysis. GOwinda effectively corrects for the gene length bias while identifying clearly over-represented GO categories, considering only SNPs being located in an exon are associated with the corresponding gene (using option: -gene-definition exon). A 5% FDR threshold was applied on GOwinda outputs. The identified GO terms in each population were clustered into homogenous groups using REVIGO (Supek et al., 2011).

## 3 Results

### 3.1 Whole Genome Diversity

The individual observed heterozygosity (*Ho*) in the five breeds was 0.28 on average, varying from 0.22 in Dman to 0.35 in Beni Guil (Table 1). The inbreeding coefficient varied thus from 0.0007 in Beni Guil to 0.095 in Dman which was the most inbred of the five Moroccan breeds.

**TABLE 1:** Number of the specific variants and neutral genetic diversity parameters, for five Moroccan breeds.

Breed parameter	Beni Guil	Dman	Ouled Jellal	Sardi	Timahdite
# Exclusive variants	341,296	1,783,651	460,461	1,621,728	934,212
Ho	0.345796	0.222807	0.313409	0.242957	0.273747
F	0.000713	0.095303	0.02129	0.026200	0.007275
Pi	0.346784	0.247173	0.321013	0.250383	0.274969

The whole genome nucleotide diversity (π), calculated over the 87 Moroccan sheep was 0.29. The Dman and Sardi breeds showed close π values of 0.247 and 0.25, respectively. Timahdite, Ouled Jellal and Beni Guil showed slightly higher π values of 0.274, 0.32 and 0.346, respectively (Table 1).

The number of breed-specific SNPs strongly varied among breeds, from 341 k and 460 k in Beni Guil and Ouled Jellal, respectively, to 1.6 and 1.78 M in Sardi and Dman, respectively (Table 1).

Regarding genetic distances, the pairwise F_ST_ was low, from 0 between Beni Guil and both Dman and Ouled Jellal to 0.004 between Sardi and both Dman and Ouled Jellal (Table 2). Furthermore, the pairwise F_ST_ between these five breeds and the cosmopolitan sheep was 0.027.

**TABLE 2:** Pairwise F_ST_ values between breeds.

Population	Beni Guil	Sardi	Ouled Jellal	Dman	Timahdite	Cosmopolitans	Wilds
Beni Guil	-					-	-
Sardi	0.002	-				-	-
Ouled Jellal	0.000	0.004	-			-	-
Dman	0.000	0.004	0.001	-		-	-
Timahdite	0.001	0.002	0.003	0.002	-	-	-
All five Moroccan breeds (87 individuals)	-	-	-	-	-	0.027	0.101

According to FLK tree (Figure S1), Timahdite is closely grouped with Sardi and Dman, Beni Guil is the most divergent, with rather long branches, among the Moroccan breeds. As expected, wilds were distant from all domestic breeds.

### 3.2 Demography of Moroccan Sheep

**FIGURE 1:**
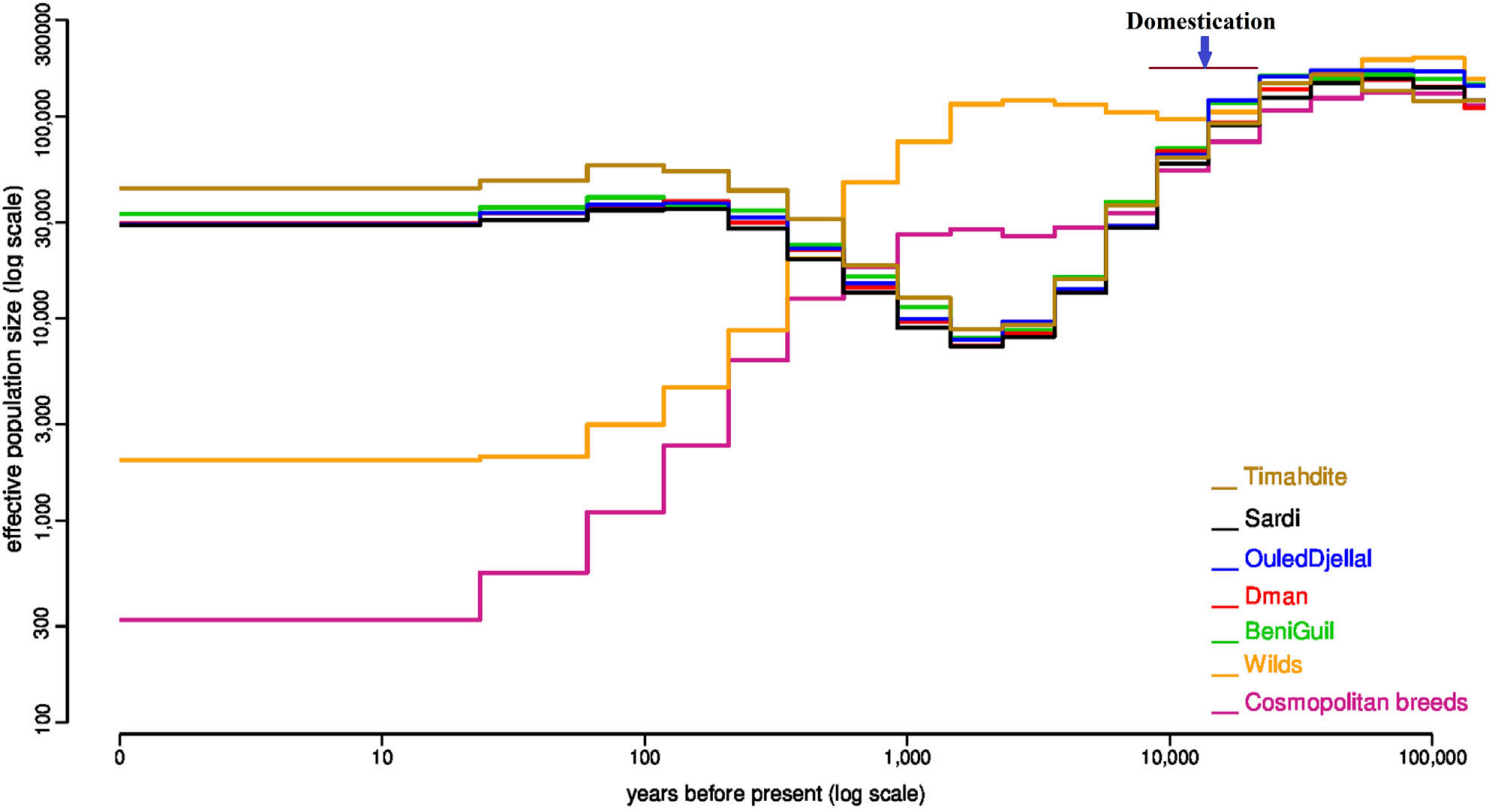
Variation of the effective population size in five Moroccan breeds in comparison with the wild Asiatic mouflon and a multi-breed group of cosmopolitan sheep. Estimates were obtained independently for each group by the popsizeABC algorithm.

The PopsizeABC analysis highlighted that before 10,000 years ago, the estimated effective population size (Ne) was similar in all populations (Figure 1). Then, the variations of Ne differed in domestic and wild animals. For domestic populations, there was a strong bottleneck probably matching the time of domestication, which was not observed for the wilds. For Moroccan sheep, the low Ne persisted until 1 K years before present, and then increased. All Moroccan breeds had a very similar trajectory, with the exception of Timahdite which showed a slightly higher *Ne* in the recent past. Conversely, in cosmopolitan sheep, the bottleneck was first less pronounced but during the last 1 K years, the Ne regularly decreased until about 300 at present. However, we do not exclude that using a pool of individuals representing several breeds may impact the accuracy of the estimates.

### 3.3 Selection Signatures

The analysis of the whole genome variants of the 87 Moroccan sheep belonging to five breeds, detected selection signatures both within (FreqHMM, Boitard et al., 2012) and between (hapFLK, Fariello et al., 2013) breeds.

#### 3.3.1 Intra-Breed Selection Signatures

We identified 182,337 SNPs in 364 genomic regions (Supplementary Table S3) under selection. Comparing the studied breeds, we found that Dman displayed the higher number of regions under selection (203) followed by Ouled Jellal (109), Sardi (96), Beni Guil (95), and Timahdite in which we identified 59 genomic regions only (Table 3).

**TABLE 3:** Number of candidate regions and SNPs under selection, with the corresponding genes, within each Moroccan sheep breed (intra-breed selection, using freqHMM).

Breed	Beni Guil	Dman	Ouled Jellal	Sardi	Timahdite
Number of regions	95	203	109	96	59
Number of SNPs	54,845	56,218	53,346	43,616	34,909
Number of genes	183	202	187	146	131

Regarding the shared selection signatures, we noticed that Dman share more SNPs/genes under selection with Sardi and Timahdite (respectively 14 K SNPs/70 genes and 9 K SNPs/60 genes; Table 4). A total of 219 SNPs were identified under selection in all five breeds, among which 203 were intergenic and 16 associated with three genes: *HMGA2* (3 SNPs), *RCOR1* (9 SNPs) and *SBF2* (4 SNPs).

**TABLE 4:** Number of selected SNPs (above the diagonal) and genes (below the diagonal) in common between two Moroccan sheep breeds (intra-breed selection, using freqHMM).

Breed	Beni Guil	Dman	Ouled Jellal	Sardi	Timahdite
Beni Guil		8,180	5,307	6,311	5,880
Dman	45		5,795	14,568	9,436
Ouled Jellal	36	44		8,238	6,846
Sardi	40	82	46		9,708
Timahdite	34	78	47	58	

#### 3.3.2 Inter-Breeds Selection Signatures

**FIGURE 2:**
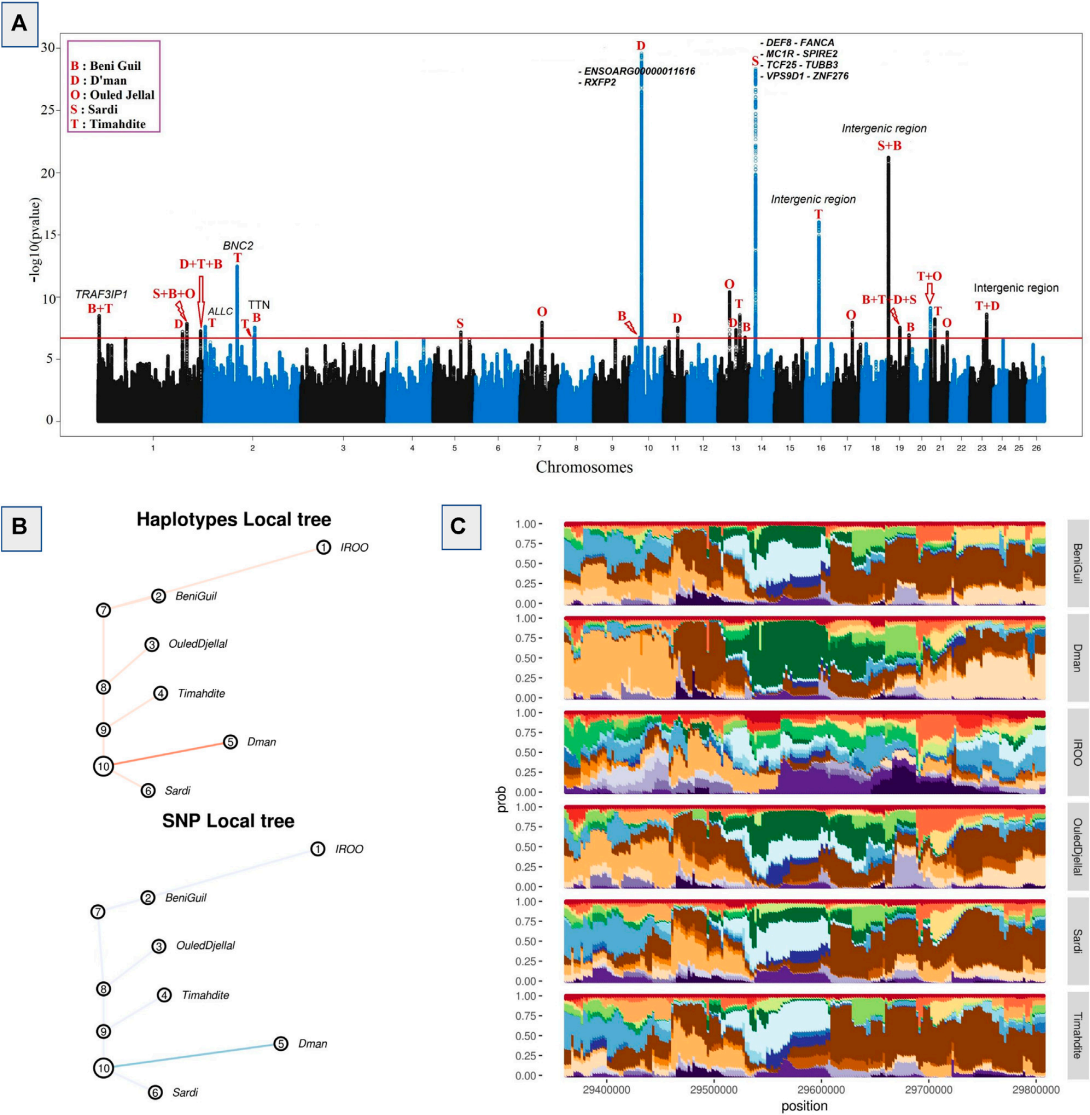
Selective sweeps detected by hapFLK and plots for an example candidate region. **(A)** Manhattan plot depicting hapFLK scores in five Moroccan sheep breeds along autosomal chromosomes. Each dot represents a SNP. The horizontal red line represents the 0.1% FDR threshold of significance. **(B)** Local tree for the example region on Chromosome 10 in Dman. **(C)** Haplotype cluster plot of the same example region on chromosome 10 in Dman (Cluster frequencies plot).

An FDR and local FDR framework was applied to the hapFLK results in order to determine a reliable selection threshold, which was set to 0.1% FDR (q-values <0.001) in order to be sufficiently conservative. The whole process identified 8,887 SNPs in 27 different regions, of which 4,131 were associated with 20 genes and 4,756 were intergenic. Among the all outliers we identified 3,908 SNPs as top candidates with *p*-values lower than 10^−12^ (Figure 2). They were distributed across five regions associated with 9 genes (of which 7 genes have 31 missense variants). Among these regions, the greatest selective sweep was located on **Chr10: 29,363,691-29,806,294**. It was related to a high differentiation between Dman and the other breeds as shown in the cluster-plot (Figure 2B) where Dman had a different haplotype from that of the other breeds, and in the local tree (Figure 2C) where Dman displayed a stronger *p*-value in both SNP local tree (FLK scores) and haplotype local tree (hapFLK scores). This selective sweep was associated with the genes *RXFP2* and *ENSOARG00000011616*. The other strongest selective sweeps were related to 1) the region located on **Chr14: 13,329,709-14,250,423** differentiating Sardi and associated to 8 genes; 2) the region located on **Chr19: 2,143,797-2,261,064** differentiating Sardi and Beni Guil from the other breeds and associated with intergenic SNPs; 3) the region located on **Chr16: 34,551,942-34,646,856** differentiating Timahdite and associated with intergenic SNPs; 4) the region located on Chr2: 84,619,111-84,759,276 differentiating Timahdite and associated to the *BNC2* gene and some intergenic SNPs (Figure 2A).

#### 3.3.3 Overall Selection Signatures

**FIGURE 3:**
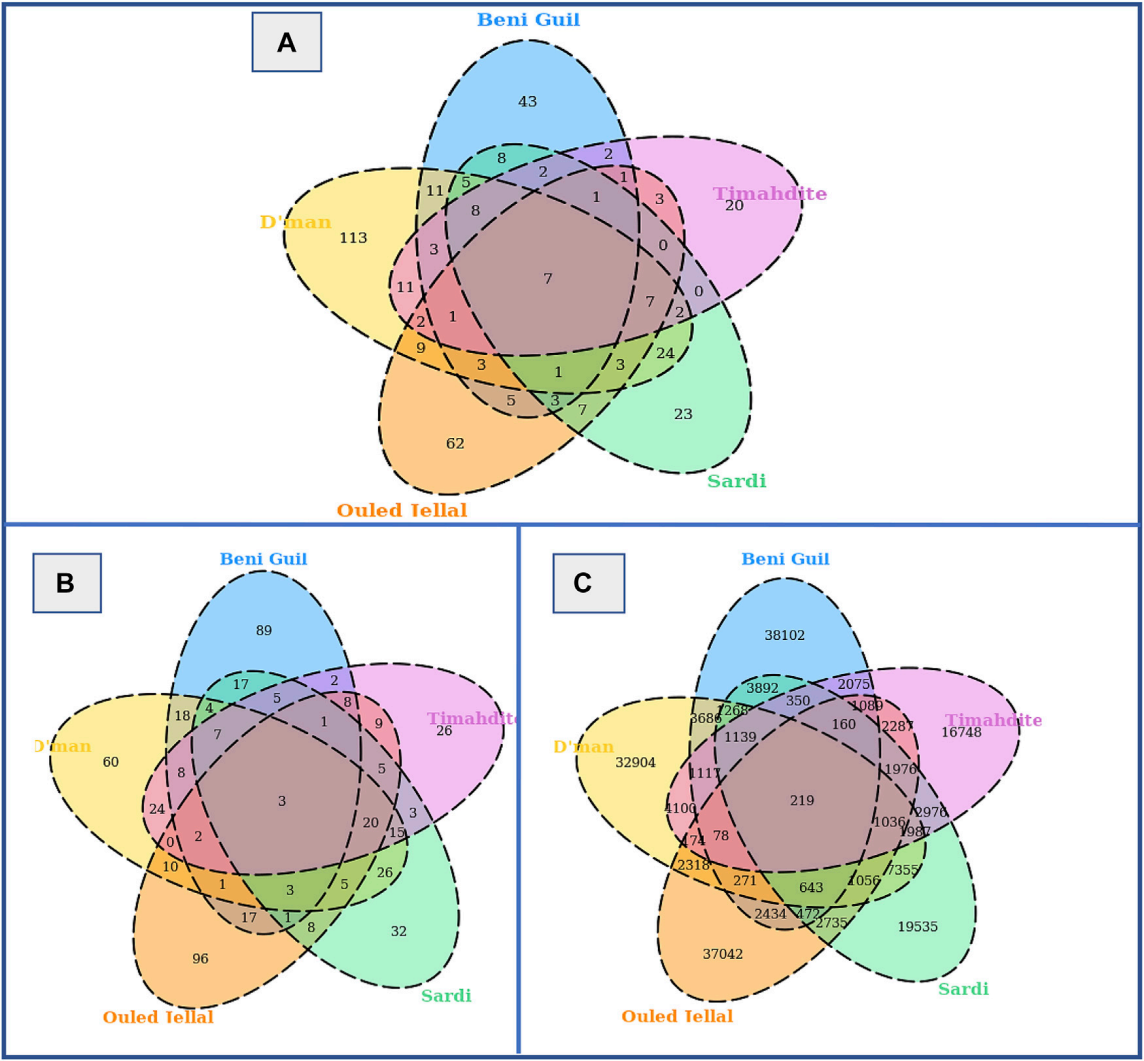
Selective sweeps, SNPs and genes identified by both freqHMM and hapFLK methods. **(A)** Venn diagram of candidate regions under selection. **(B)** Venn diagram of genes under selection. **(C)** Venn diagram of SNPs under selection.

When combining both approaches, from a total of 31,721,507 analyzed SNPs, we identified as putatively under selection 46,799 variants associated with 155 genes in Sardi, 37,511 SNPs associated with 138 genes in Timahdite, 59,351 variants associated with 206 genes in Dman, 56,995 associated with 186 genes in the Beni Guil breed and 53,990 variants associated with 189 genes in Ouled Jellal. The Venn diagrams in Figure 3 illustrate the number of regions, SNPs and associated genes that are specific or common to the studied breeds. We identified 219 SNPs within 7 genomic regions under selection in all 5 breeds, which are associated with 3 genes: *HMGA2*, *RCOR1* and *SBF2*.

##### 3.3.3.1 Biological Processes Targeted by Selection

The selection signatures detected by both methods (i.e., FreqHMM & HapFLK) allowed identifying a total of 7,735 GO categories enriched in genes under selection (Table 5), based on a threshold of FDR<5% (Supplementary Tables S4–S7). The lowest number of enriched GO categories per breed was for Timahdite, where 1,465 categories clustered in 223 homogenous groups of biological processes. The Sardi breed exhibited the highest number of enriched GO term, with 1,611 categories clustering in 229 groups of processes. In regard to these high numbers of enriched biological processes, we’ll limit our discussion to those which roles are the most straightforward given the related phenotypic traits and husbandry practices.

**TABLE 5:** Examples of biological processes enriched in candidate genes in the five sheep breeds.

GO term	Biological process	Candidate genes associated	*p*-value	Associated breed
GO:0070344	Regulation of fat cell proliferation	PID1	0.0000688058	Beni Guil
GO:0098743	Cell aggregation	COL11A1	0.0000688058	Beni Guil
GO:0008544	Epidermis development	BNC1	0.0000741624	Dman
GO:0007292	Female gamete generation	FSHR BNC1	0.0000741624	Dman
GO:0048469	Cell maturation	RXFP2	0.0000741624	Dman
GO:0009650	UV protection	SDF4	0.0000667761	Ouled Jellal
GO:0043112	Receptor metabolic process	SH3GLB1 LMBRD1	0.0000667761	Ouled Jellal
GO:1901568	Fatty acid derivative metabolic process	FADS1 PLA2G10	0.0000679420	Sardi
GO:0043586	Tongue development	BNC2	0.0000692810	Timahdite
GO:0040007	Growth	BNC2 ESR1	0.0000692810	Timahdite
GO:0060749	Mammary gland alveolus development	ESR1	0.0000692810	Timahdite
GO:0007631	Feeding behavior	MRAP2	0.0000692810	Timahdite

## 4 Discussion

### 4.1 Whole Genomic Diversity

The heterozygosity measured in Moroccan breeds was generally moderate (mean of 0.28) and globally lower than the values reported for Iranian sheep breeds (Eydivandi et al., 2021) and Welsh breeds (Beynon et al. (2015). Dman and Sardi breeds have even much lower heterozygosity values. Our results showed low inbreeding in Moroccan sheep, comparably to those reported by Eydivandi et al. (2021) except for Dman which was the most inbred (*F* = 0.1). F_ST_ values showed no clear differentiation between Moroccan breeds with an average of ∼0.002. These F_ST_ values are lower than those found in Welsh local sheep (Beynon et al., 2015) but still comparable to that of Russian local sheep (Deniskova et al., 2018), which were both estimated from WGS data.

Based on nucleotide diversity (π), we found that Dman was the most diversified of the Moroccan breeds. This could be explained by a lower intensity of selection, a higher founder population and wider breeding area for this breed. The slightly higher inbreeding in Dman would be explained by its sub-structuration in isolated Oases that leads to the mating of closely related individuals and by the longstanding use reproductive rams (Bouix and Kadiri, 1974; Darfaoui, 2000). However, these levels are still much lower than those measured by Beynon et al. (2015) on Welsh sheep breeds.

Furthermore, the past demographic dynamics (Figure 1), inferred with PopsizeABC and associating a pool of wild animals, was consistent with the occurrence of a domestication event 10,500 years ago in the Fertile Crescent (Vigne, 2011; Demirci et al., 2013). Indeed, earlier than 10.5 K years ago, effective sizes of all wild and domestic populations were similar, which is consistent with a common origin from the same ancestral species of *Ovis Orientalis* (Vigne, 2011). Around 10 K years B.P, the strong bottleneck linked to domestication is observed in all domestic breeds, Moroccan and cosmopolitan (Figure 1). The lasting decline in Moroccan breeds sizes between around 10 K and 2 K years would correspond to a long-term migration process consisting in colonizing gradually new constraining environments. Indeed, the colonization process lasted for millennia (Taberlet et al., 2008) and Morocco, which is located at the end of several migration routes, was marked by two main arrivals of domestic sheep; the first one occurred around 8,600 years B.P and would be associated with the first Berbers who settled in Morocco, while the second originated from Iberia around 7,100 years B.P (kandoussi et al., 2020).

Between 2,000- and 1,000-years B.P, all Moroccan breeds gradually increased in size which corroborates their common history and the absence of isolation obetween breeds at that time. The Timahdite breed shows a higher increase in effective population size for about five centuries in comparison with the other breeds. Inversely, the industrial breeds show a continuous decline during the last millennium which has been accentuated during the last century. Similarly, the effective size of the wild mouflon gradually declined for about a millennium, which is in line with the degradation of their habitat by humans and the intensification of hunting activity (Rezaei, et al., 2010). More recently, over the last 700 years, the *Ne* of Moroccan and cosmopolitan breeds evolves in different ways with a huge drop of the cosmopolitan populations, which is consistent with the evolution of breeding practices which can go as far as the intensive use of artificial insemination. Finally, if we except Timahdite which behaved differently for the last 500 years, the other Moroccan breeds remain very close, which suggests their recent foundation.

Our estimates of the current *Ne* (Supplementary Table S1) and diversity parameters (Table 1) would illustrate the uniqueness of Moroccan local sheep breeds. The current *Ne* values for domestics (highest for Timahdite with Ne ∼ 44 K) are very high when compared to what is reported in the literature for sheep (e.g., Maiwashe and Blackburn, 2004; Tapio et al., 2005; Beynon et al., 2015), while the estimates for mouflon populations (Ne ∼ 2 K) are comparable to those reported by Beynon et al. (2015) in Welsh domestic sheep. We should however consider that estimates for recent time using popsize ABC can sometimes reach 5 to 10 times those based on pedigree or molecular data. This has been observed in a cattle breed (Boitard et al., 2016b). These results confirm actual measures of diversity using metrics such as nucleotide diversity. In any case, these results would also show the strong adaptive potential of Moroccan sheep breeds and the opportunity of implementing efficient programs for their breeding while maintaining this richness. Indeed, a high effective population size would be associated to a high intra-breed genomic diversity that include both adaptive and standing genetic variation. Furthermore, the latter is known to be a main driver of new adaptive traits that can be needed/useful in the context of new environmental pressures (Savolainen et al., 2013). Inversely, the very low effective size (Ne = 317) estimated from a mix of cosmopolitan breeds, despite their presence in very large numbers worldwide, show the huge threat they represent if their use to replace local breeds continues (Taberlet et al., 2008).

### 4.2 Selection Signatures in Moroccan Sheep

The selection signatures identified would shed light on the biological processes underlying both adaptive and zootechnical traits selected in each breed. Most of the regions identified under selection were intergenic (64%). This would illustrate the important role of regulation in the realization of biological processes and the expression of traits as reported by ENCODE Project Consortium et al. (2007). However, this could sometimes be due to hitch-hiking mechanisms and also to some limitations in the functional annotation of genomes (Benjelloun et al., 2015).

From the literature, the three genes identified as under selection in all five studied breeds (i.e., *HMGA2*, *RCOR1* and *SBF2*) are generally involved in cellular functioning. The expression of *HMGA2* is strongly associated with body size and growth in mice, humans and dogs (Webster et al., 2015; Vignali and Marracci, 2020; Yang et al., 2010). Furthermore, the inactivation of *HMGA2* in pigs resulted in a huge body-size reduction (Chung et al., 2018). The *RCOR1* gene has a role in transcriptional regulation, and is involved in repressing neuronal gene expression in non-neuronal cells (Coulson, 2005). Mutations in the *SBF2* gene were associated with autosomal recessive Charcot-Marie-Tooth Disease type 4B2 in humans (Senderek et al., 2003), and has been associated with growing traits in cattle (Jahuey-Martínez et al., 2016) and horse (Al Abri et al., 2018).

Besides, we identified many selection signatures (589 candidate regions) specific to one or a few breeds. Several GO Terms were enriched in genes putatively selected (Supplementary Tables S4–S8); they were associated with large categories such as: organ development, pigmentation pathways, proliferation and lipid metabolism. We discuss here the genes which role appears to be quite straightforward, which includes participation to functional processes related to morphology, pigmentation or skin coloration, zootechnical performance and prolificacy.

### 4.3 Sardi Breed

The *MC1R* gene, which is candidate in the Sardi breed, has been associated with a large panel of skin or coat colors (Sturm and Duffy, 2012). It may be involved in the particular coloration pattern specific to this breed and preferred by consumers (Supplementary Figure S1C) in relation to the religious ceremony of Aid Al Adha. This breed is characterized by a white head devoid of wool with black spots around the eyes, muzzle, paws (feet) and at the tips of the ears (Boujenane 1999, http://www.anoc.ma/les-races/races-ovines/sardi/, January 2021). Another candidate specific of Sardi is *SLC9A3*. The deficiency of this gene causes severe obstructive azoospermia and infertility in male mice (Wang et al., 2017).

### 4.4 Timahdite Breed

The *BNC2* and *EDN3* genes, identified in Timahdite, have been reported as potentially associated with skin pigmentation (Hider et al., 2013; Fariello et al., 2014), and may be involved in the specific skin coloration of this breed (Supplementary Figure S1D): a brown head, without spots neither black nor yellow, always very clear, sometimes, and extending behind the ears and into the trough area (Boujenane, 1999).

We also identified *ESR1* as a candidate, which is a major mediator of estrogen action and is strongly linked to bone mass and osteoporosis in mice (Nakamura et al., 2007; Börjesson et al., 2010). Some alleles were significantly associated with adult human height (Wood et al., 2014). This gene would play a role in growth, as well as another candidate, *MRAP2,* which modulates melanocortin receptor signaling and have been associated with severe obesity in human (Asai et al., 2013; Schonnop et al., 2016).

### 4.5 Dman Breed

*RXFP2* has been associated with the horned/polled phenotype in many sheep breeds (Johnston et al., 2011; Dominik et al., 2012; Kijas et al., 2012; Wang et al., 2014). This gene was identified under selection only in Dman which is the only Moroccan breed (Supplementary Figure S1E) with both polled males and females (Boujenane 2006; Boujenane et al., 2013). Also, the seasonal expression of this gene was correlated with the differentiation of Leydig cells in the testis (Hombach-Klonisch et al., 2004; Serranito et al., 2021), which would suggest a possible involvement in fertility. Similarly, *BNC1* and *FSHR* were identified, while Dman is known for its exceptional prolificacy in comparison with the other Moroccan breeds. It has an ovulation rate of 2.8 versus a maximum of 1.3 in the other breeds, with the ability to breed all year long with 190–350 days of interval between two lambings (Boujenane, 2006) depending on husbandry practices. It is also characterized by its early puberty (210 days in average, Boujenane 2006). The *FSHR* gene would be related to fertility, as the protein it encodes for is located in the testis and granulosa cells of the ovaries (Kwok et al., 2005), and is involved in follicle maturation and proliferation of granulosa cells (Sudo et al., 2002). Also, mutations in this gene were associated with the Polycystic Ovary Syndrome in humans which is characterized by obesity and anovulatory infertility (Gu et al., 2010; Laven, 2019). Besides, *BNC1* is a candidate which encodes for a zinc finger protein present, among other places, in the basal cell layer of the epidermis and in hair follicles and which is also expressed in the germ cells of testis and ovary (Luchi and Green, 1999). This protein is thought to play a regulatory role in keratinocyte proliferation and has been shown to be involved in premature ovarian failure and testicular premature aging (Zhang et al., 2018; Li et al., 2020). It is a crucial transcription factor for spermatogenesis and male fertility (Li et al., 2020). Thus, we could hypothesize thus that these last two genes play a role in the reproductive performance of the Dman breed.

### 4.6 Beni Guil Breed

The *TTN* gene was identified under selection (with 5 missense variants) exclusively in the Beni Guil breed. It has been associated with meat and carcass traits in pigs (Braglia et al., 2013), meat colour, pH and conductivity in loin 24 h postmortem (Wimmers et al., 2007). Similarly, *PID1* is identified as a candidate. This gene modulates insulin signaling and mitochondrial function in adipocytes and muscle cells, and was reported as a candidate gene for fat deposition, in humans, based on its high expression in adipose tissue of obese subjects in comparison with normal subjects (Wang et al., 2006). These two genes may be responsible for meat quality and the important fat percentage known of Beni Guil. Indeed, this breed is one of the best meat breeds in Morocco. Its carcass scores a high quality and a high fatness state with a white and firm cover fat, what makes it well appreciated by professionals and consumers (Belhaj et al., 2020). Since 2011, it has been certified by the PGI (Protected Geographical Indication) label (Belhaj et al., 2018), which represents the excellence of European agricultural food production.

### 4.7 Ouled Jellal Breed

The *SDF4* (also called *CAB45*) found in Ouled Jellal was involved in protecting against UV-induced damages (Zhu et al., 2008) and was revealed as a modulator of cell proliferation and tumor growth (Chen et al., 2016). Mutations in this gene may have occurred, as an adaptation, in response to the effect of sunlight (i.e., exposure to Ultraviolets) on the entirely white skin color of the Ouled Jellal breed (Chellig, 1992). in fact, fair/clear types of skin color are found, in humans, to be significantly more sensitive to UV rays than darker skin types (Halder and Bridgeman-Shah 1995; Hemminki et al., 2002).

### 4.8 Common Selection Signatures

Besides specific-breed candidates, the Microphthalmia-associated transcription factor (*MITF*) is a selected gene in both Beni Guil, Dman, Sardi and Timahdite. It is a basic transcription factor which regulates the differentiation and development of melanocytes and pigment cell-specific transcription of the melanogenesis enzyme genes (Saravanaperumal et al., 2014). This gene was also associated with eye and coat spotting color in some dog breeds (Rothschild et al., 2006; Stritzel et al., 2009). We could hypothesize that this gene may interact with other genes to produce different coat color patterns.

## 5 Conclusion

We characterized the neutral diversity, demographic history and selection signatures using whole genome variants in the five main sheep breeds from Morocco. Globally, these five breeds are not very genetically differentiated, but they show a high number of specific variants and very high effective population sizes unlike cosmopolitan breeds. This illustrates that Moroccan indigenous breeds are highly diversified and have thus the potential to develop key adaptive characteristics to face upcoming climate changes. This makes them valuable resources for conservation and future preservation of sheep even at the worldwide scale.

Investigations on selection signatures provided valuable insights about genes and biological processes targeted by selection, which are essentially involved in pigmentation, zootechnical performance, adaptation and reproduction traits. Many genomic regions highlighted here need further investigations to decipher their specific roles in the expression of phenotypes specific to the studied breeds. However, the outlier genomic variants identified here represent valuable candidates and should be conserved in priority when conceiving the upcoming genomic breeding programs. Those programs have to consider improving production as well as adaptation traits while maintaining the diversity of these local sheep breeds.

## Data Availability

Publicly available datasets were analyzed in this study. This data can be found here: ftp://ftp.ebi.ac.uk/pub/databases/nextgen/ovis/variants/genus_snps/.
