# Supplementary material for: Genomic Uniqueness of Local Sheep Breeds From Morocco
Source: Front Genet. 2021 Dec 2;12:723599. doi: 10.3389/fgene.2021.723599 (PMC8675355; doi:10.3389/fgene.2021.723599)
Supplement: Supplementary file 1 [file DataSheet1.PDF]

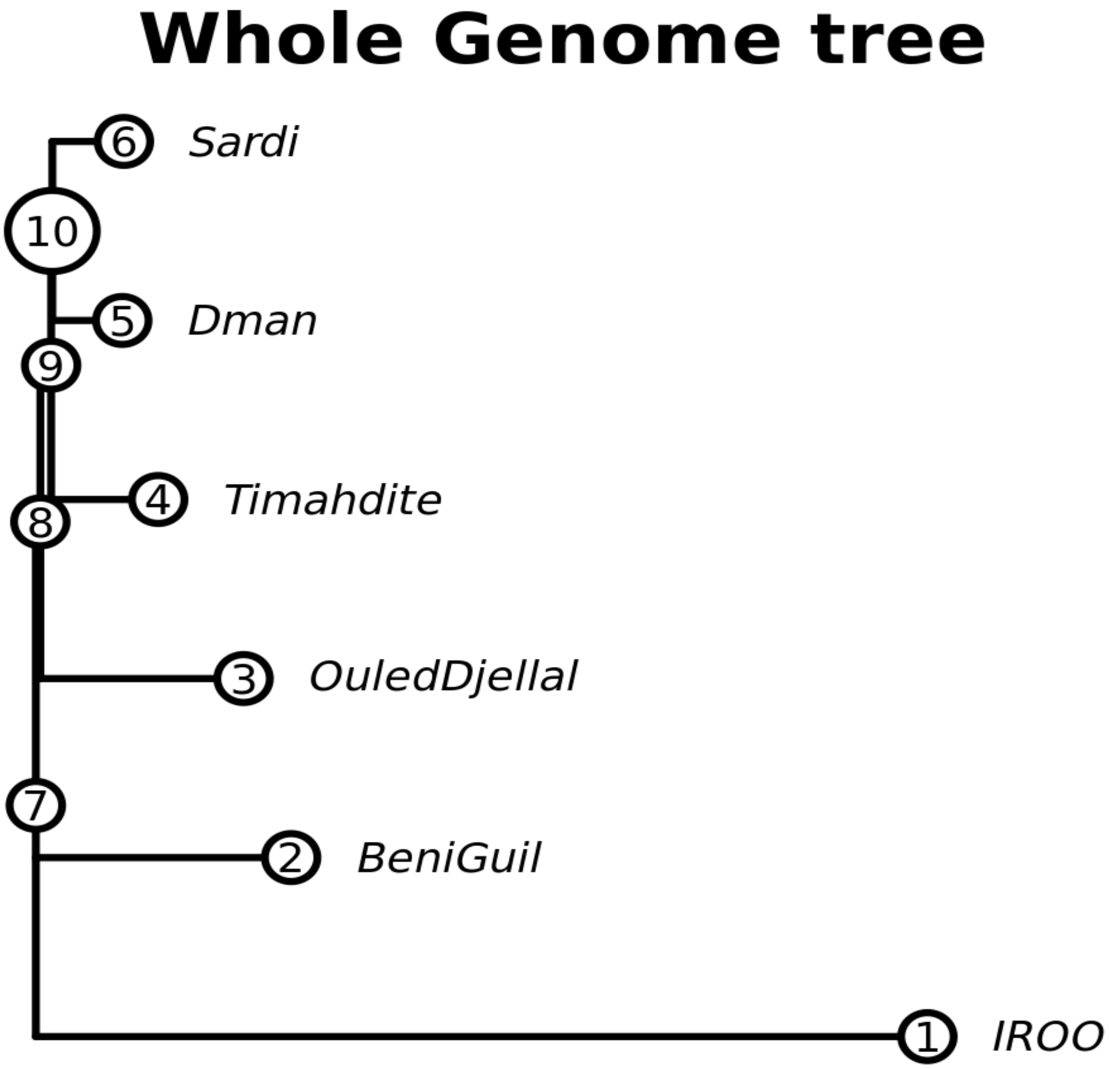

Figure S1: Whole genome tree of the analyzed groups estimated by FLK

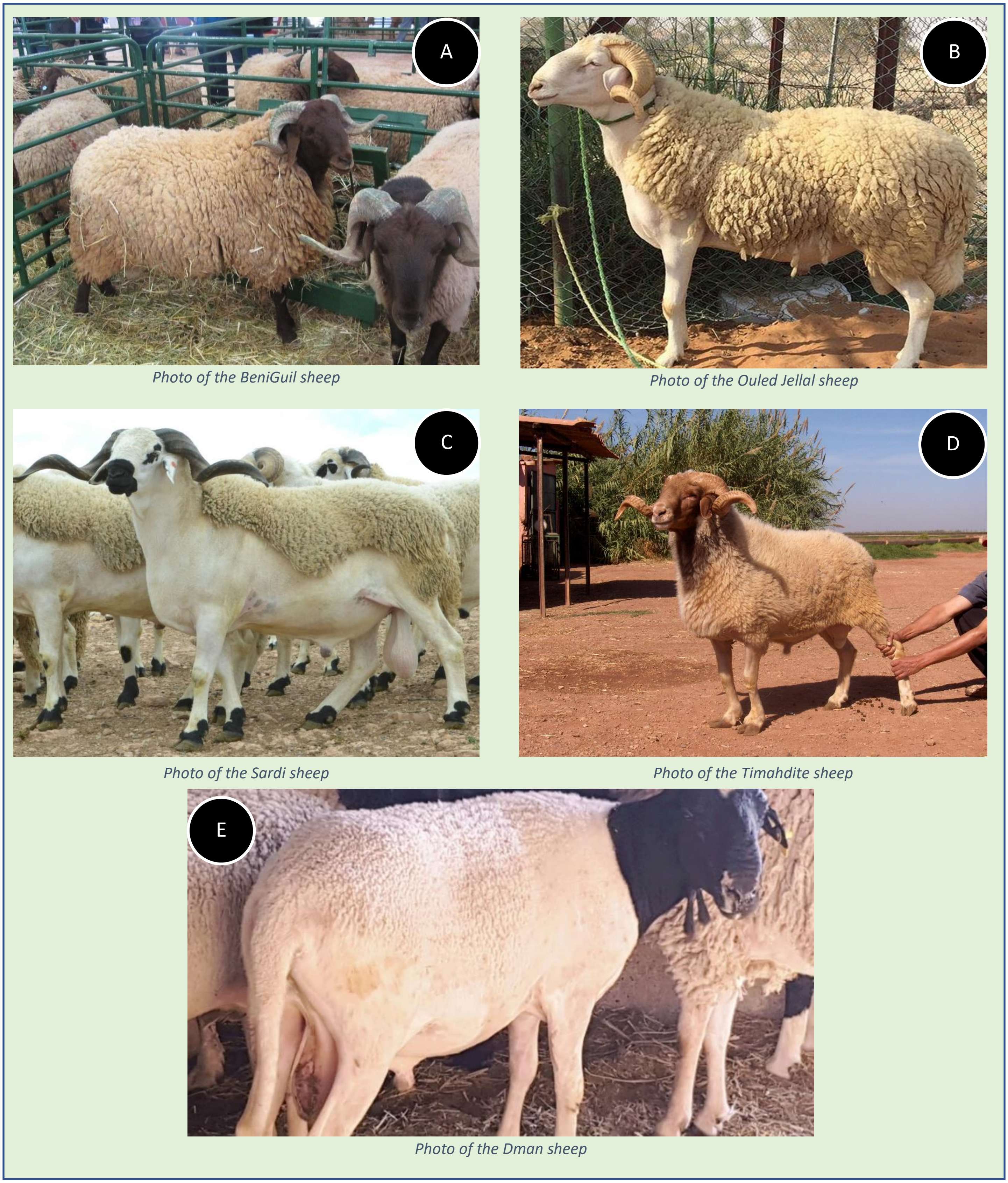

Photo of the BeniGuil sheep

Photo of the Ouled Jellal sheep

Photo of the Sardi sheep

Photo of the Timahdite sheep

Photo of the Dman sheep

Figure S2: Sheep phenotypes for each of the five Moroccan breeds

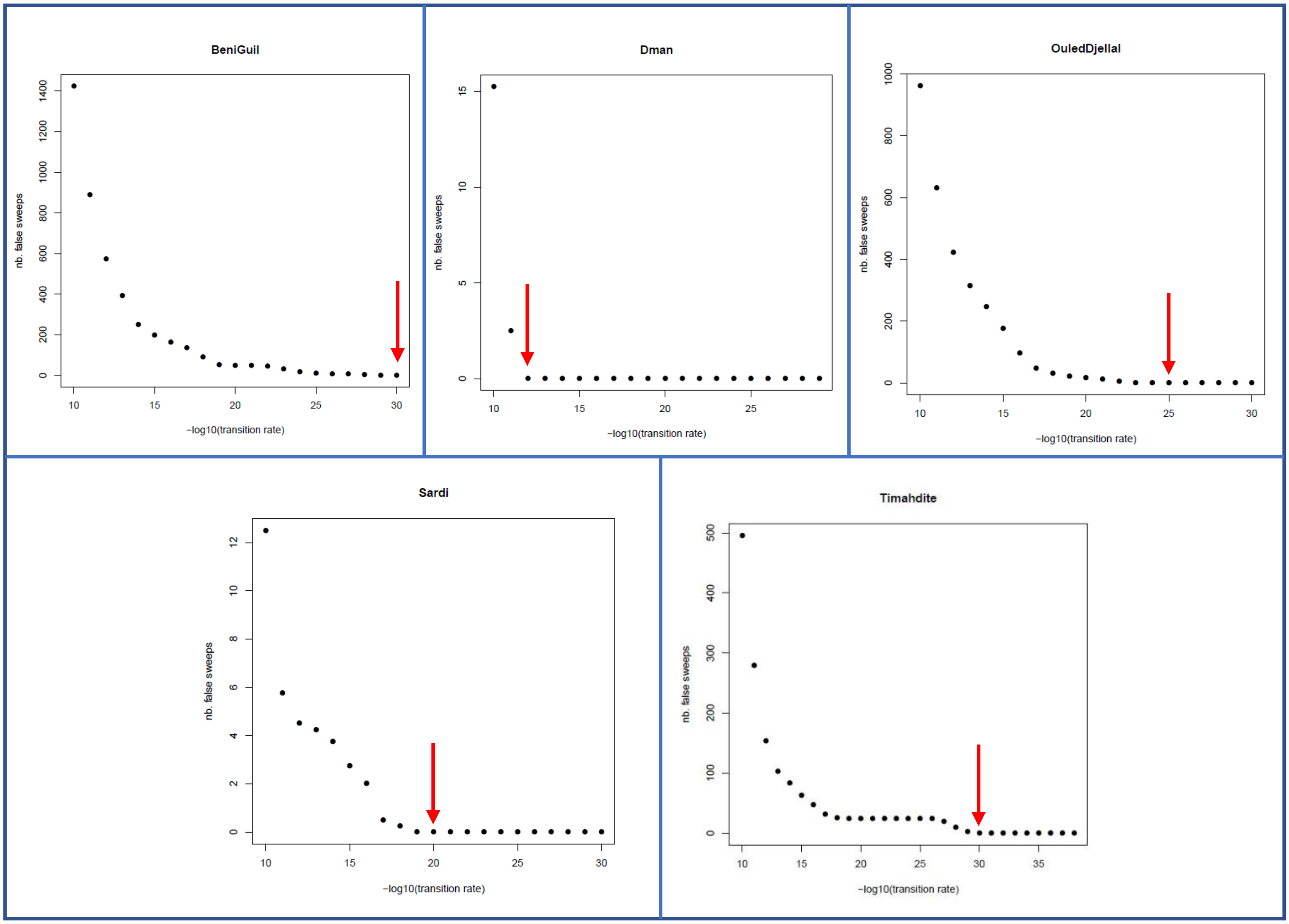

Figure S3: False sweeps control test for choosing K parameter (sensitivity of freqHMM) for each population. The red arrow shows the chosen K value (when the number of false sweeps tends to Zero)

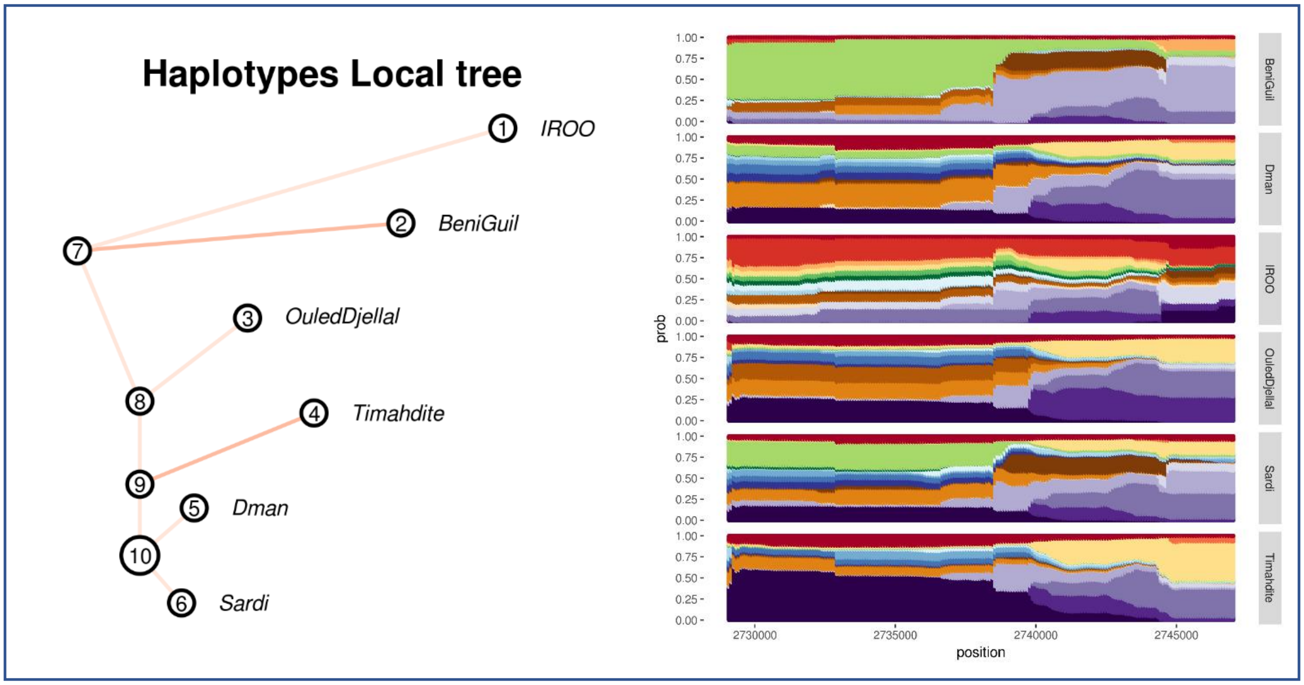

Figure S4: Haplotype local tree (left) and Haplotype cluster plot (right) of the region Chr1:2729994-2746603, showing the most differentiated breeds: BeniGuil and Timahdite.

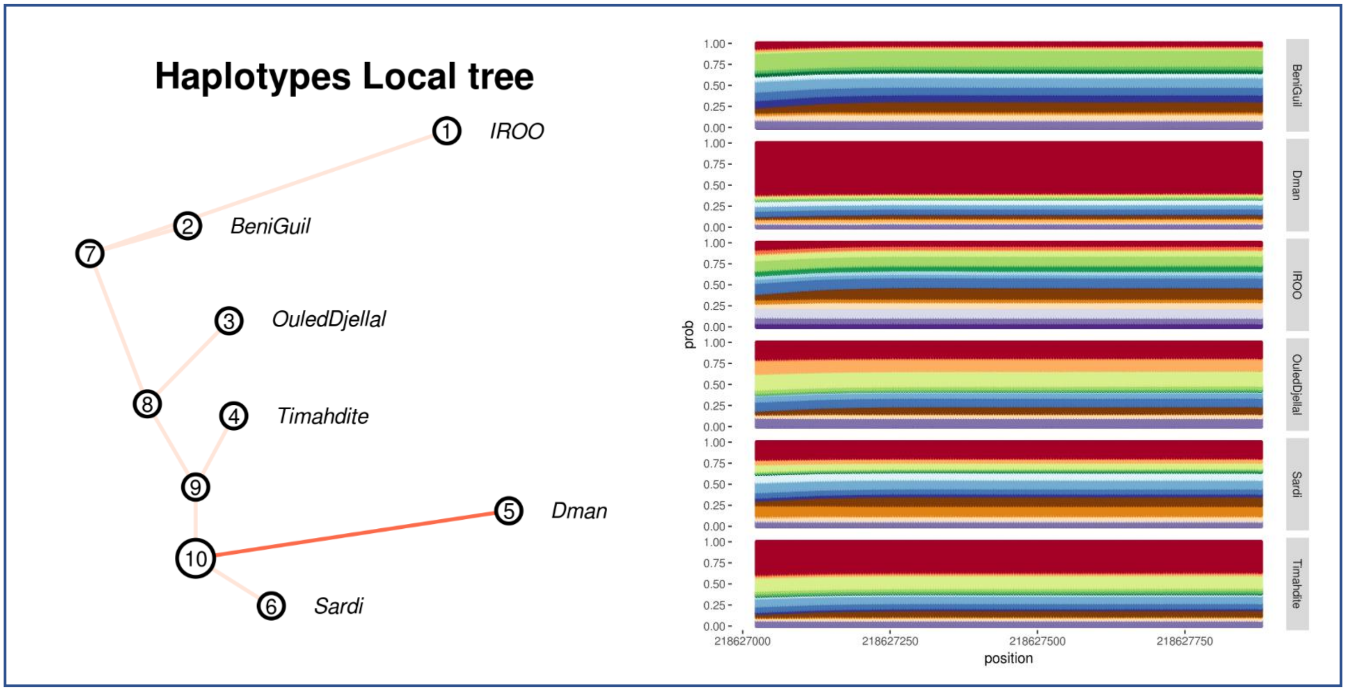

Figure S5: Haplotype local tree (left) and Haplotype cluster plot (right) of the region Chr1:218627080-218627670, showing the most differentiated breed: Dman.

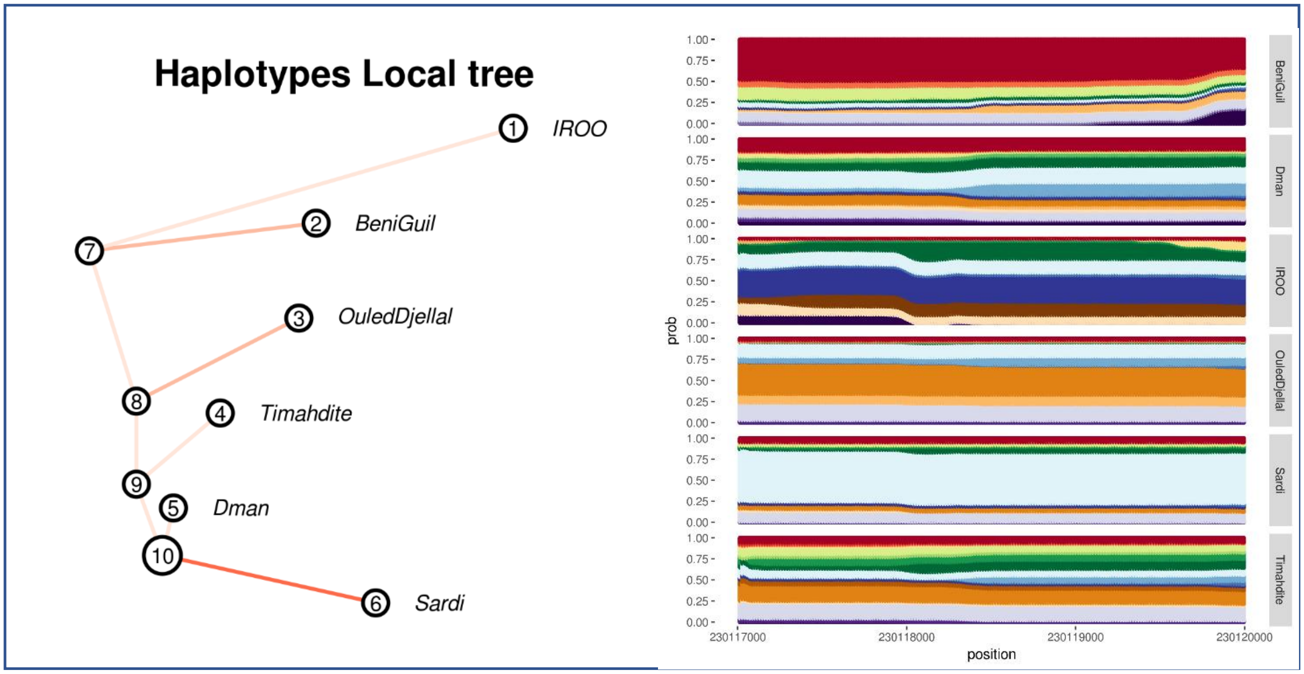

Figure S6: Haplotype local tree (left) and Haplotype cluster plot (right) of the region Chr1:230117531–230119919, showing the most differentiated breeds: BeniGuil, Ouled Jellal and Sardi.

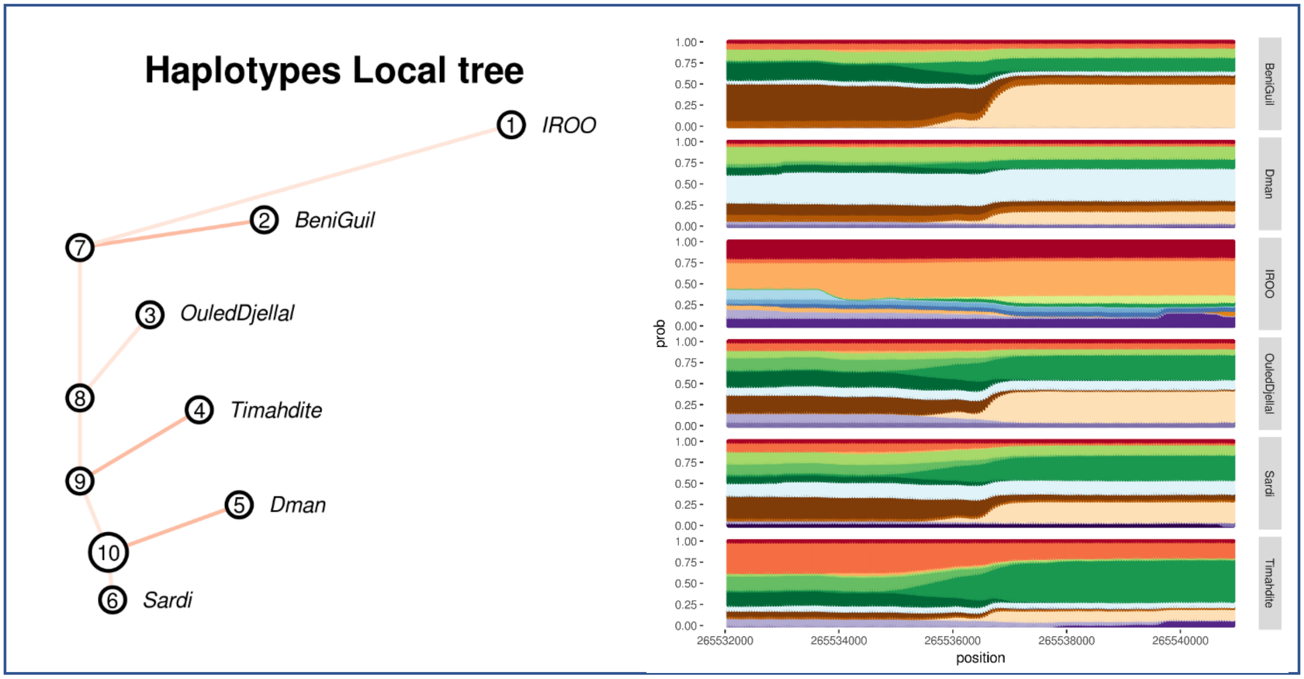

Figure S7: Haplotype local tree (left) and Haplotype cluster plot (right) of the region Chr1:265532246–265540828, showing the most differentiated breeds: BeniGuil, Dman and Timahdite.

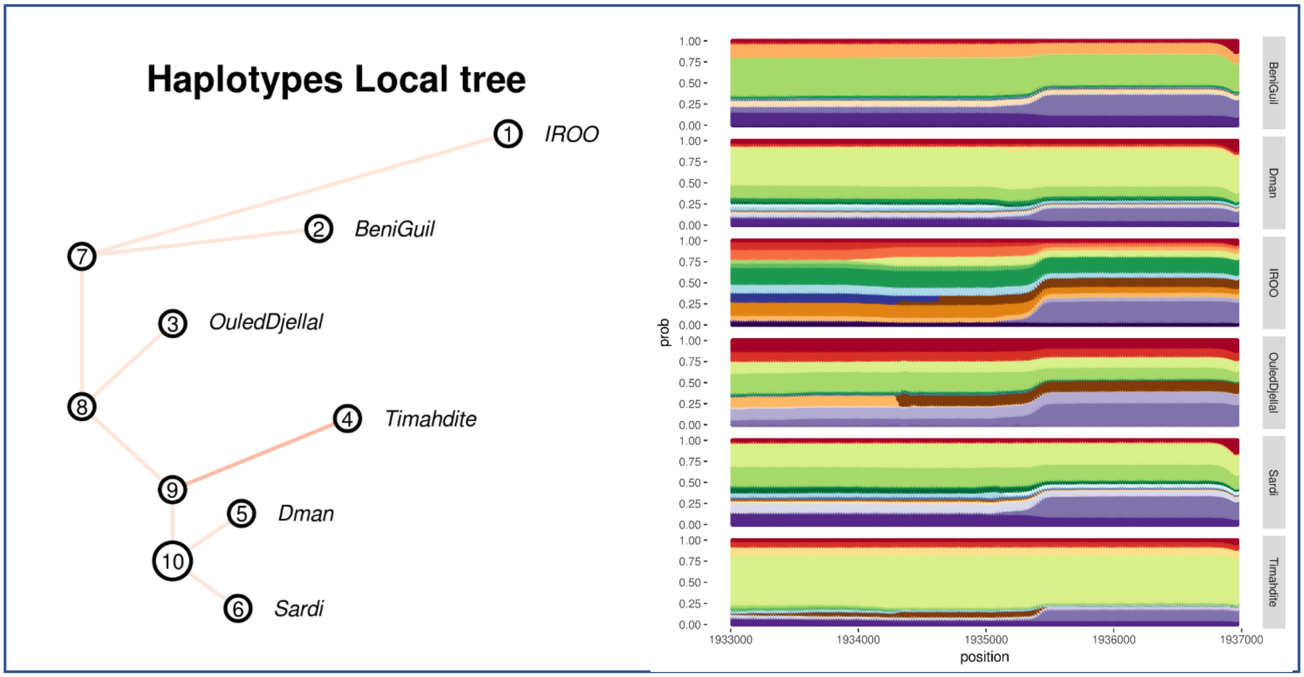

Figure S8: Haplotype local tree (left) and Haplotype cluster plot (right) of the region Chr2:1933721–1936398, showing the most differentiated breed: Timahdite.

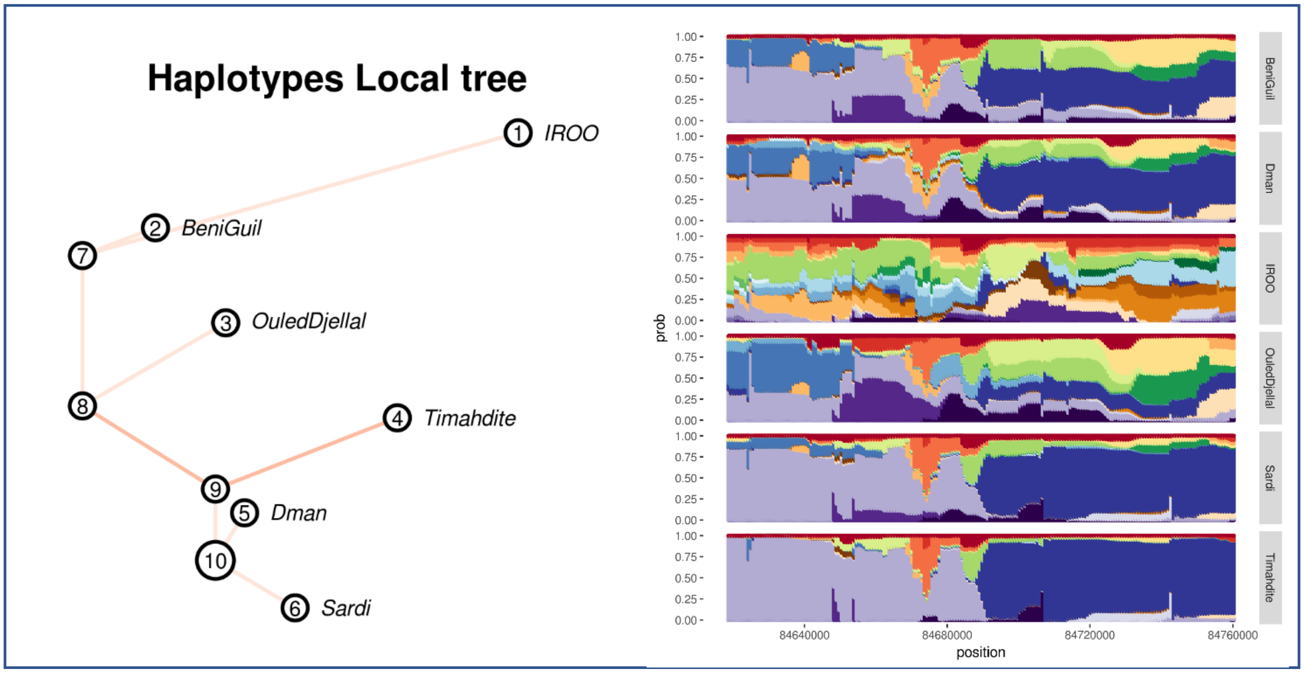

Figure S9: Haplotype local tree (left) and Haplotype cluster plot (right) of the region Chr2:84619111–84759276, showing the most differentiated breed: Timahdite.

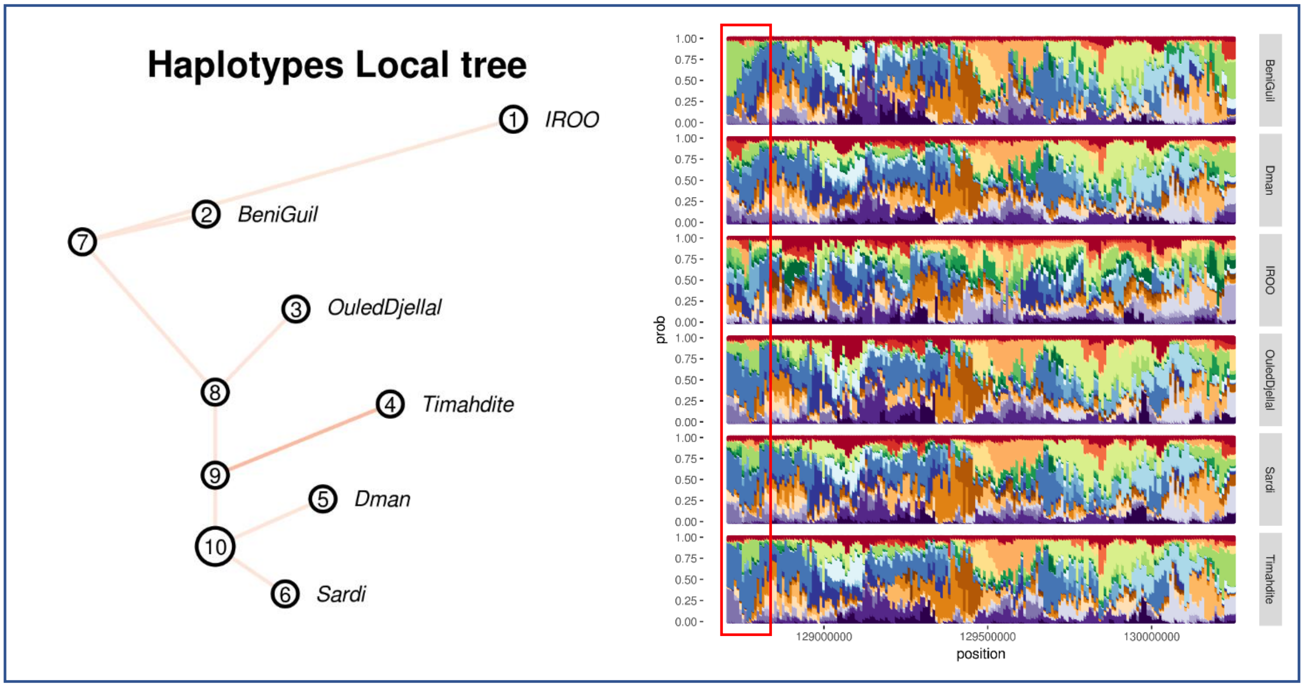

Figure S10: Haplotype local tree (left) and Haplotype cluster plot (right) of the region Chr2:128712616-128713554, showing the most differentiated breed: Timahdite.

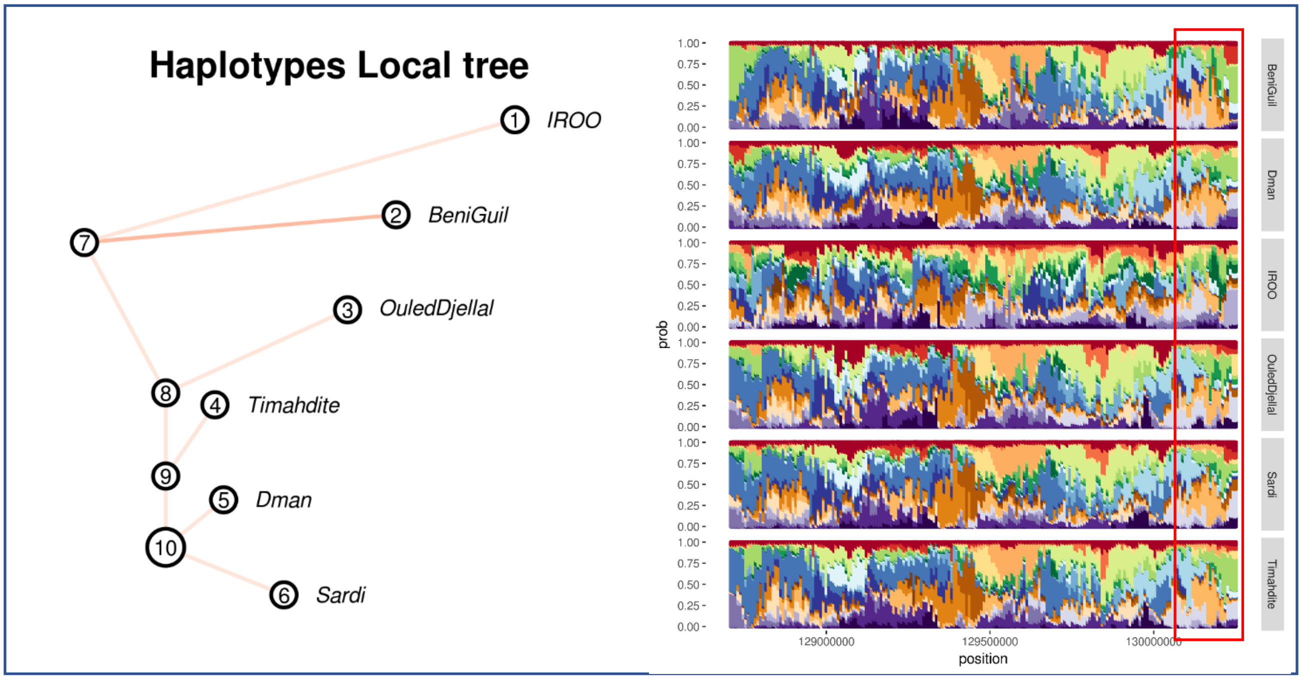

Figure S11: Haplotype local tree (left) and Haplotype cluster plot (right) of the region Chr2:130242281-130247334, showing the most differentiated breed: BeniGuil.

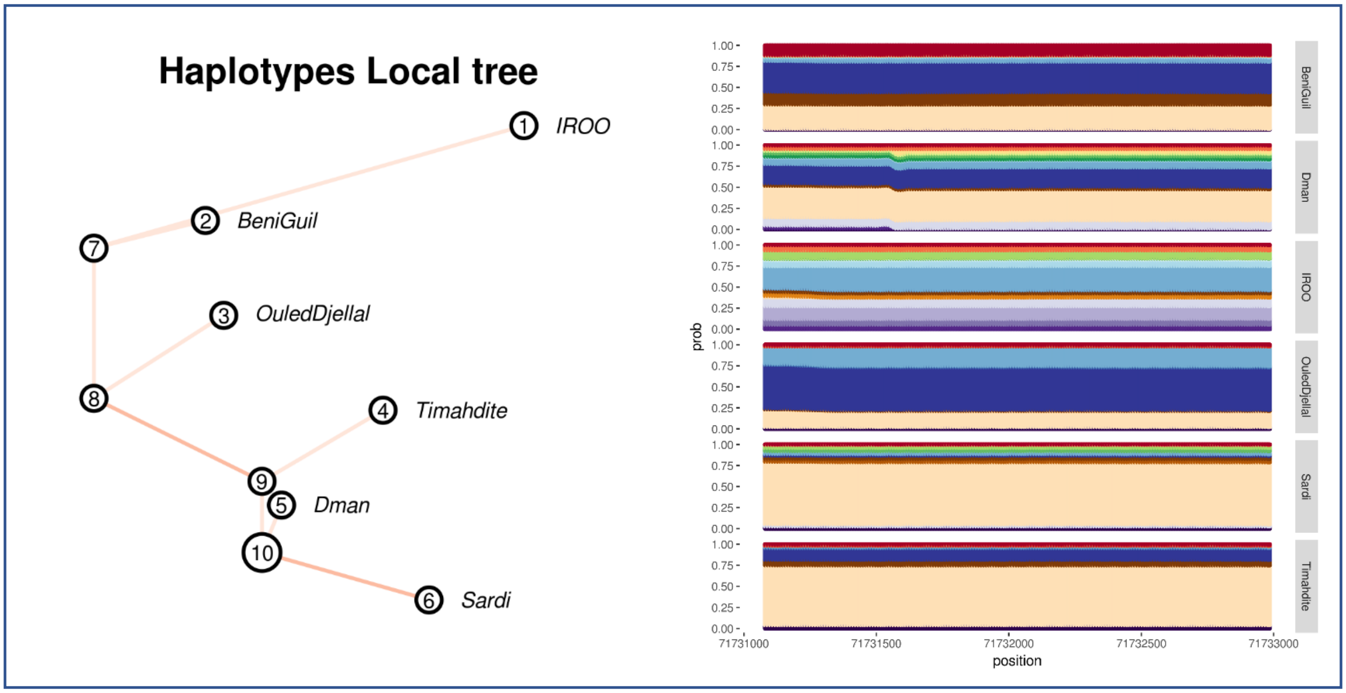

Figure S12: Haplotype local tree (left) and Haplotype cluster plot (right) of the region Chr5:71731270–71732981, showing the most differentiated breed: Sardi.

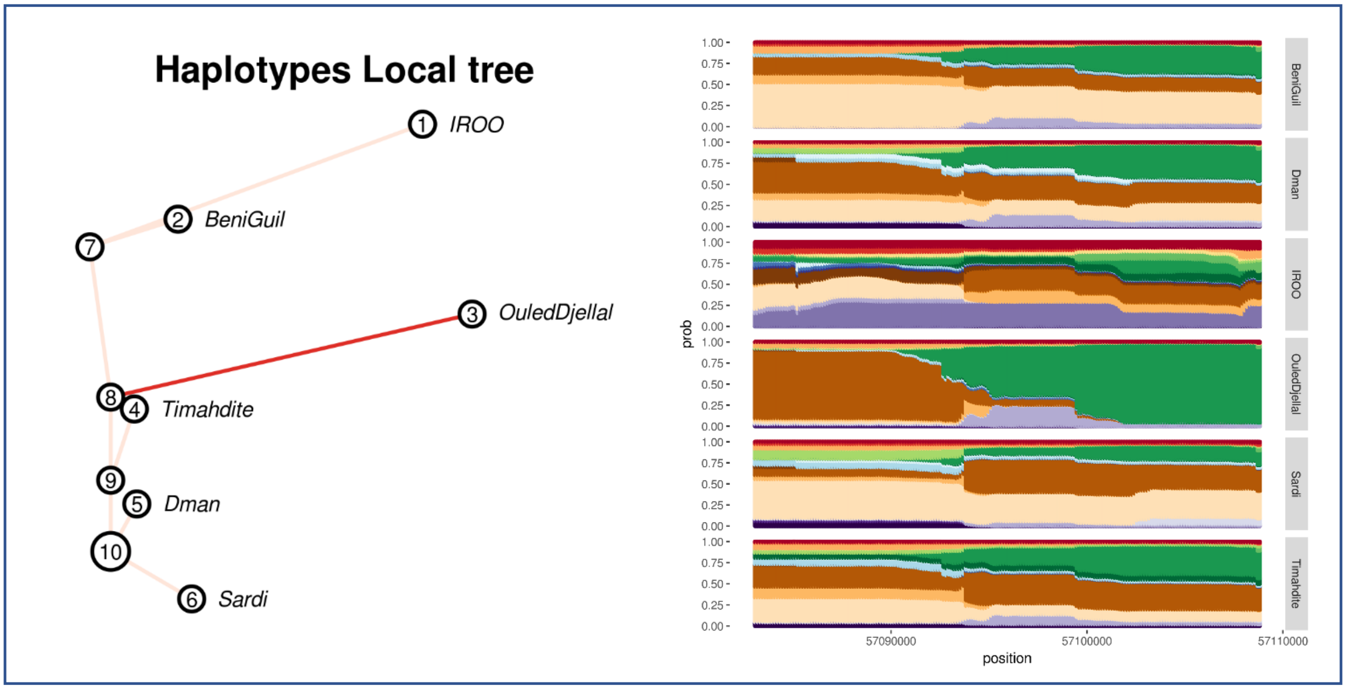

Figure S13: Haplotype local tree (left) and Haplotype cluster plot (right) of the region Chr7:57083691-57108734, showing the most differentiated breed: Ouled Jellal.

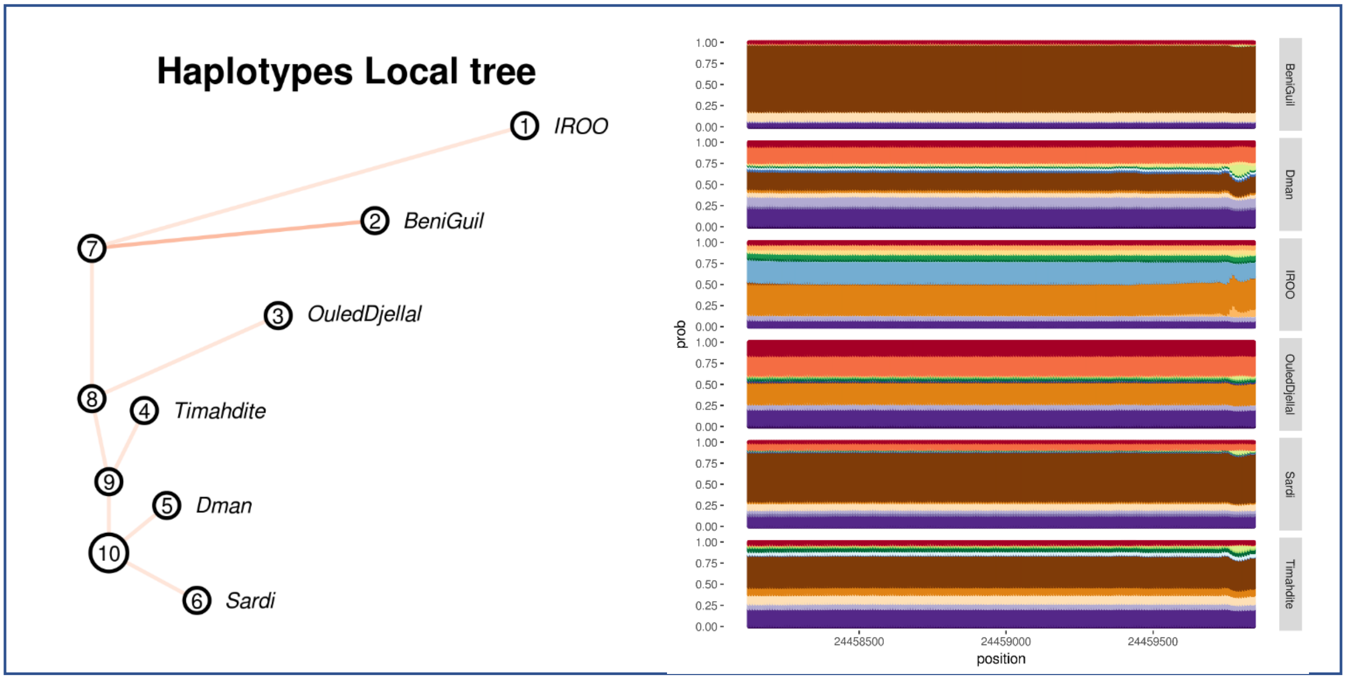

Figure S14: Haplotype local tree (left) and Haplotype cluster plot (right) of the region Chr10:24458234-24459169, showing the most differentiated breed: BeniGuil.

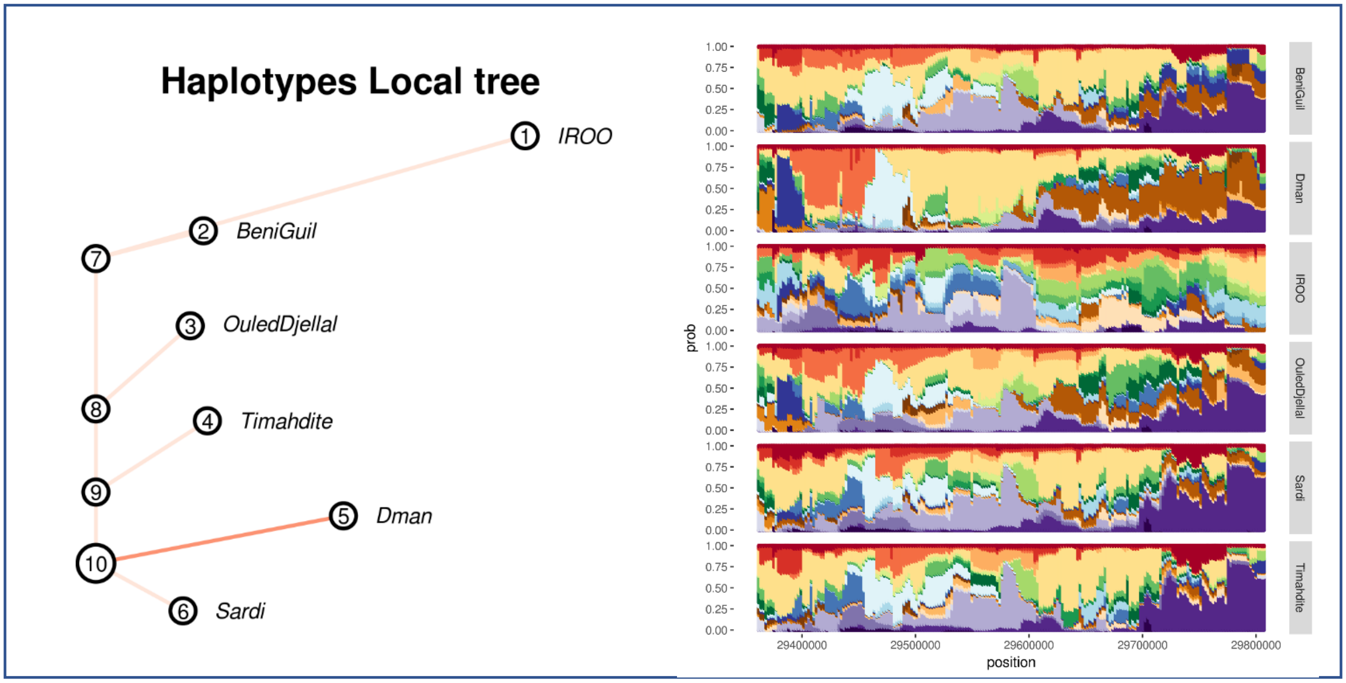

Figure S15: Haplotype local tree (left) and Haplotype cluster plot (right) of the region Chr10:29363691-29806294, showing the most differentiated breed: Dman.

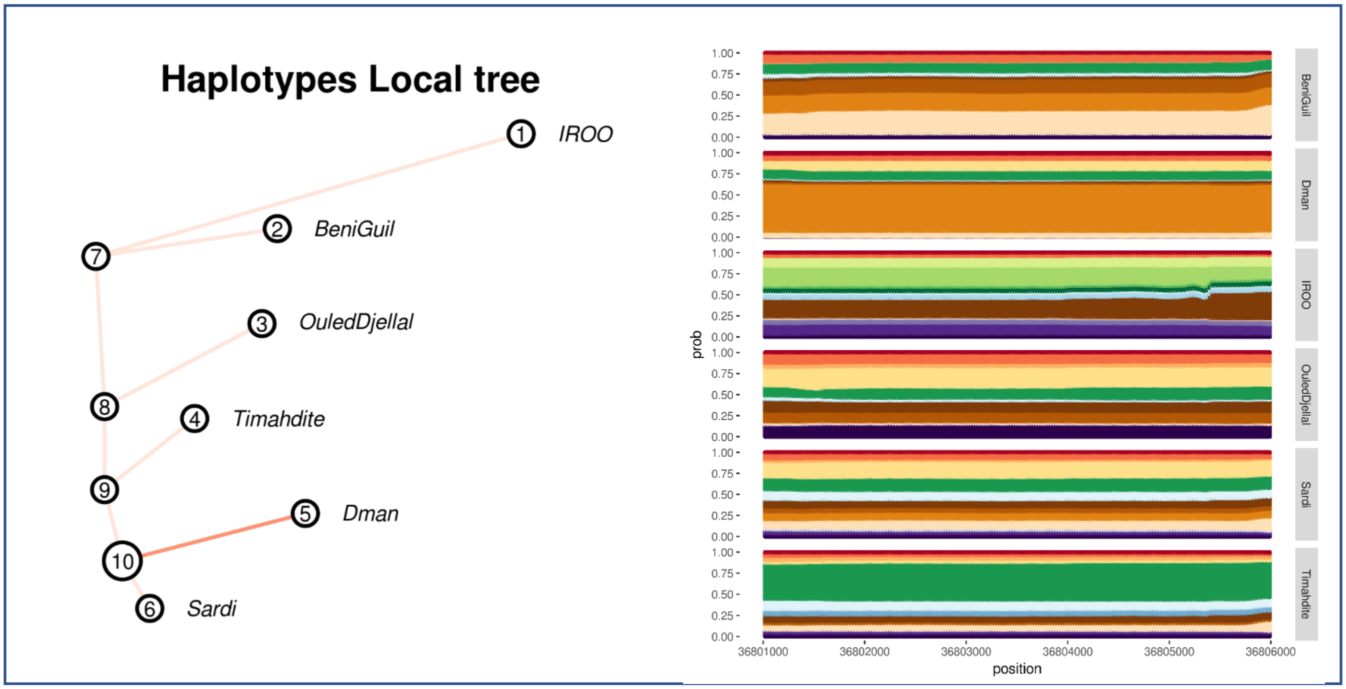

Figure S16: Haplotype local tree (left) and Haplotype cluster plot (right) of the region Chr11:36801443-36805430, showing the most differentiated breed: Dman.

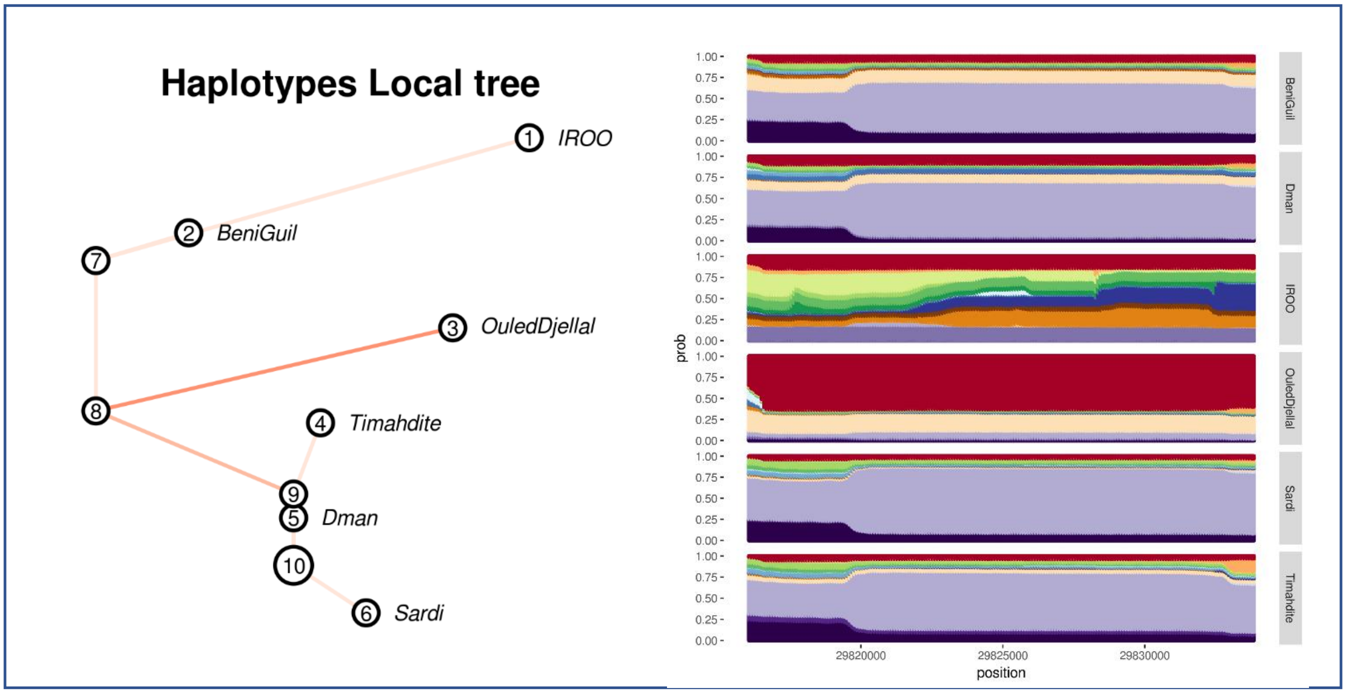

Figure S17: Haplotype local tree (left) and Haplotype cluster plot (right) of the region Chr13:29816181-29833006, showing the most differentiated breed: Ouled Jellal.

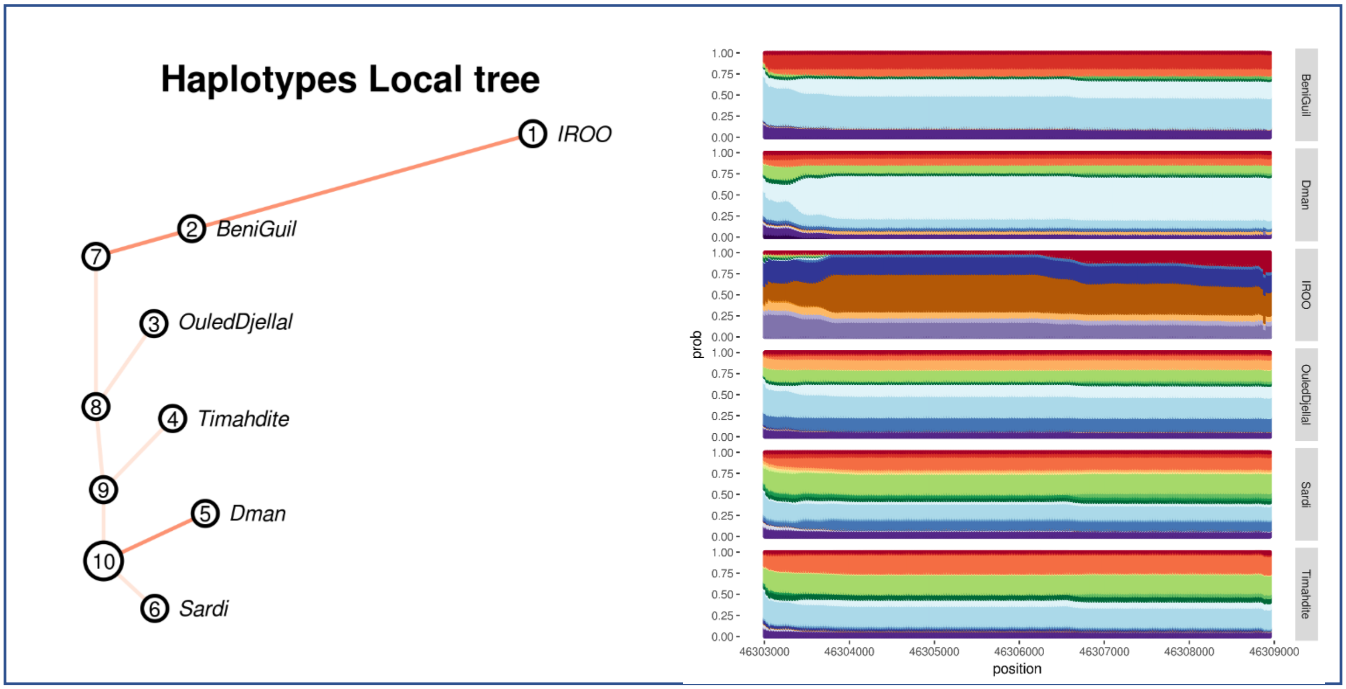

Figure S18: Haplotype local tree (left) and Haplotype cluster plot (right) of the region Chr13:46303939-46308888, showing the most differentiated breed: Dman.

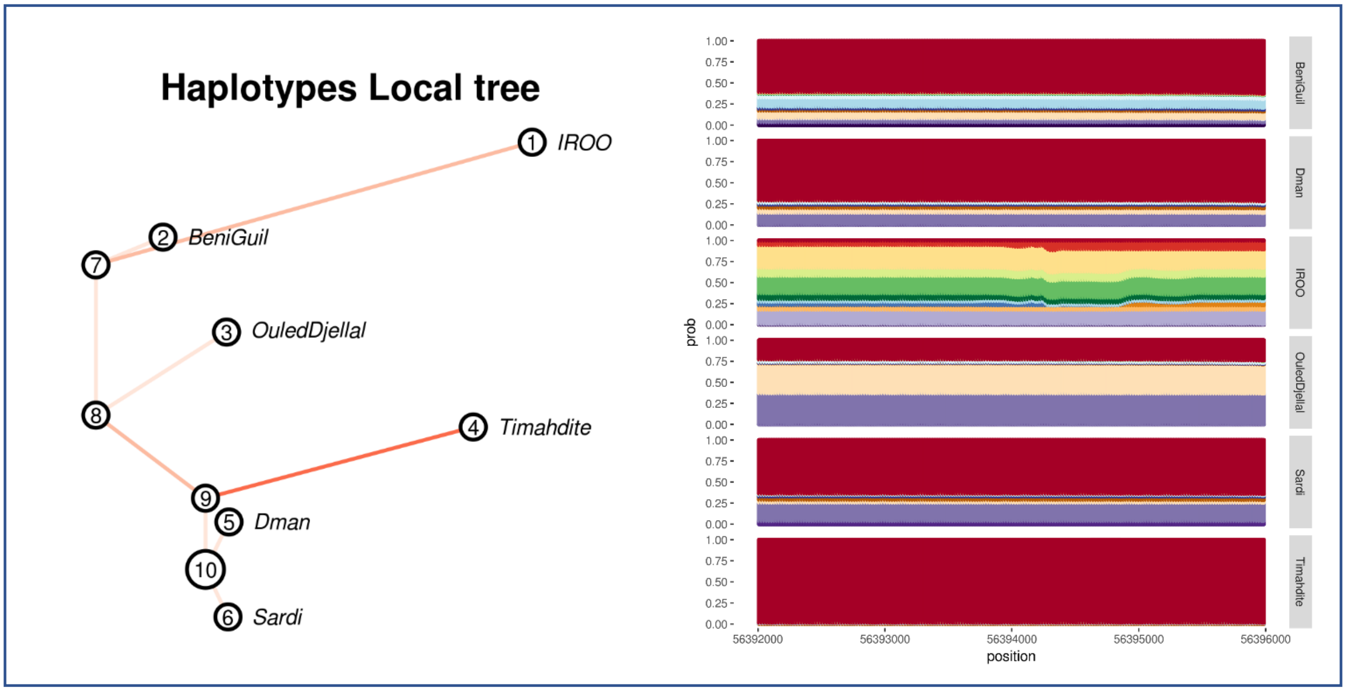

Figure S19: Haplotype local tree (left) and Haplotype cluster plot (right) of the region Chr13:56392318-56395466, showing the most differentiated breed: Timahdite.

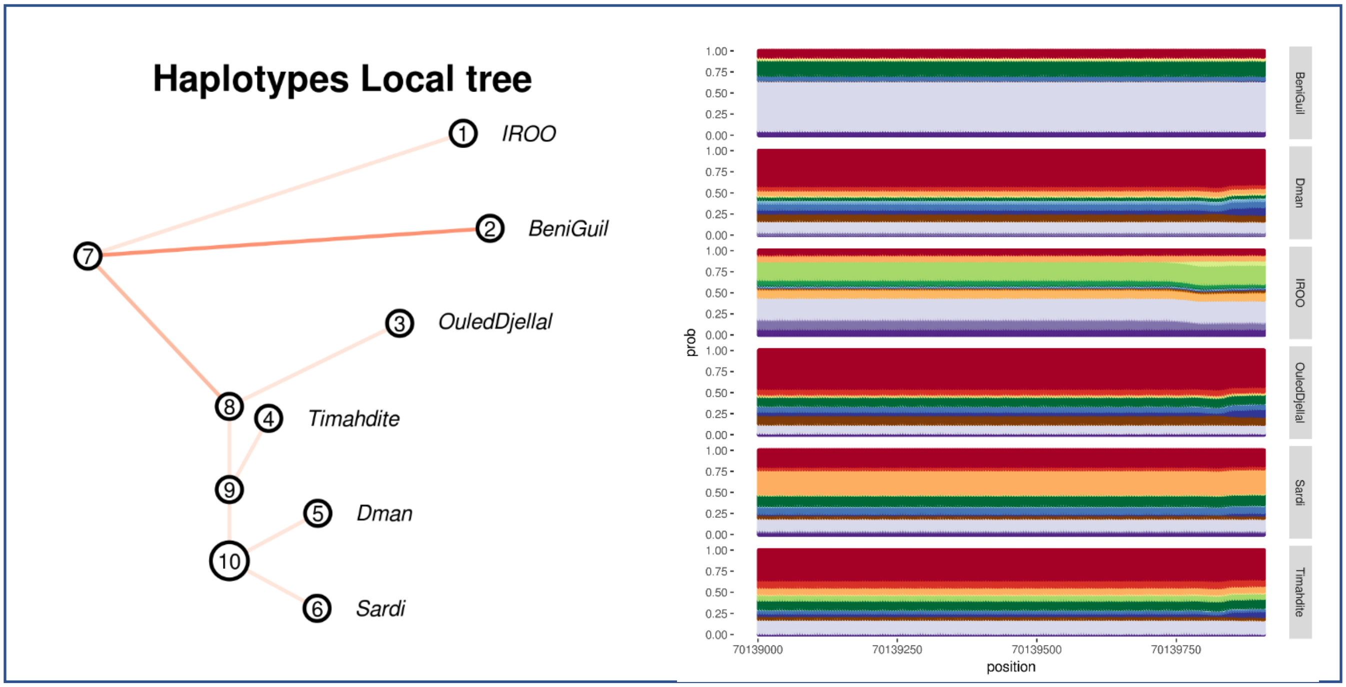

Figure S20: Haplotype local tree (left) and Haplotype cluster plot (right) of the region Chr13:70139004-70139391, showing the most differentiated breed: BeniGuil.

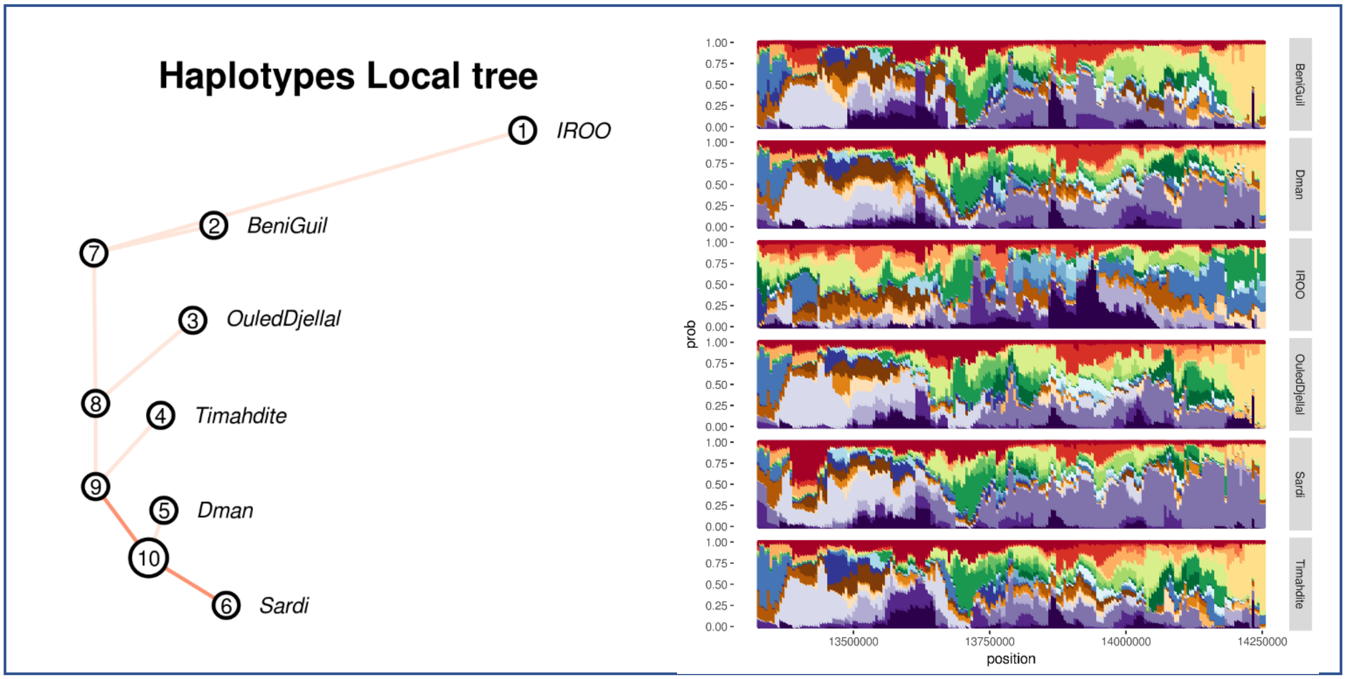

Figure S21: Haplotype local tree (left) and Haplotype cluster plot (right) of the region Chr14:13329709-14250423, showing the most differentiated breed: Sardi.

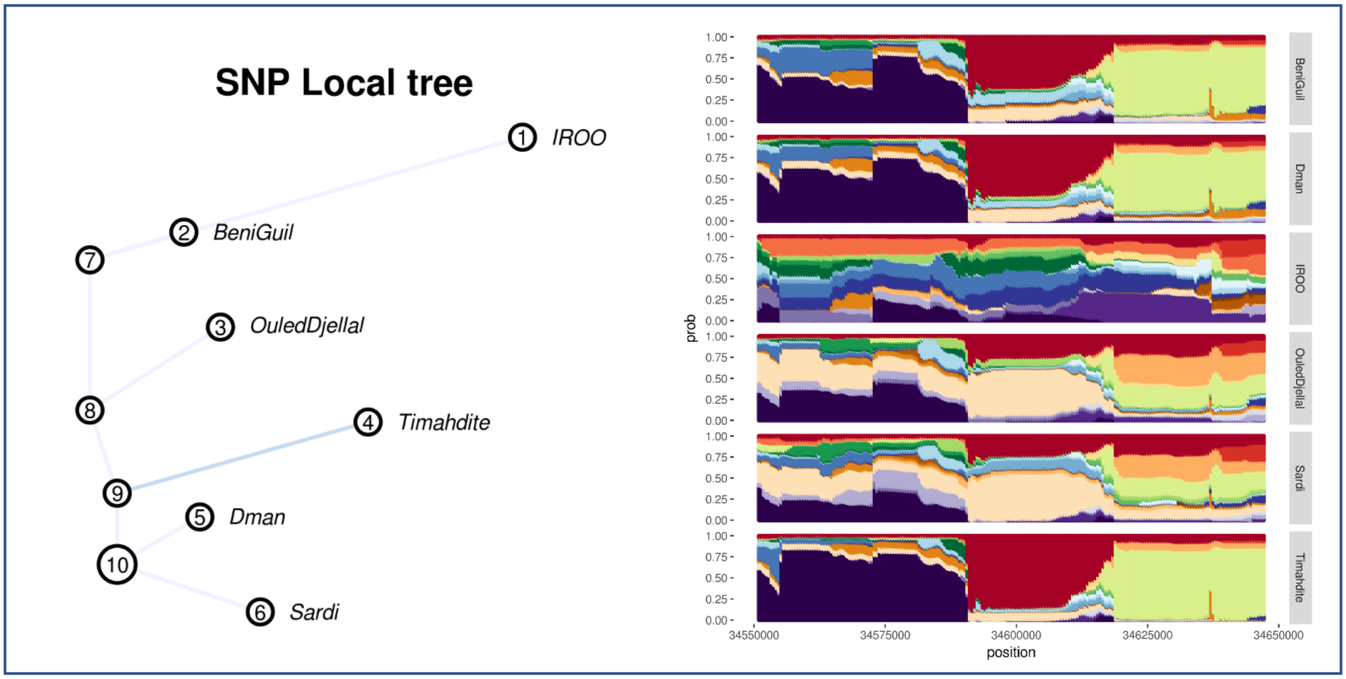

Figure S22: SNP local tree (left) and Haplotype cluster plot (right) of the region Chr16:34551942-34646856, showing the most differentiated breed: Timahdite.

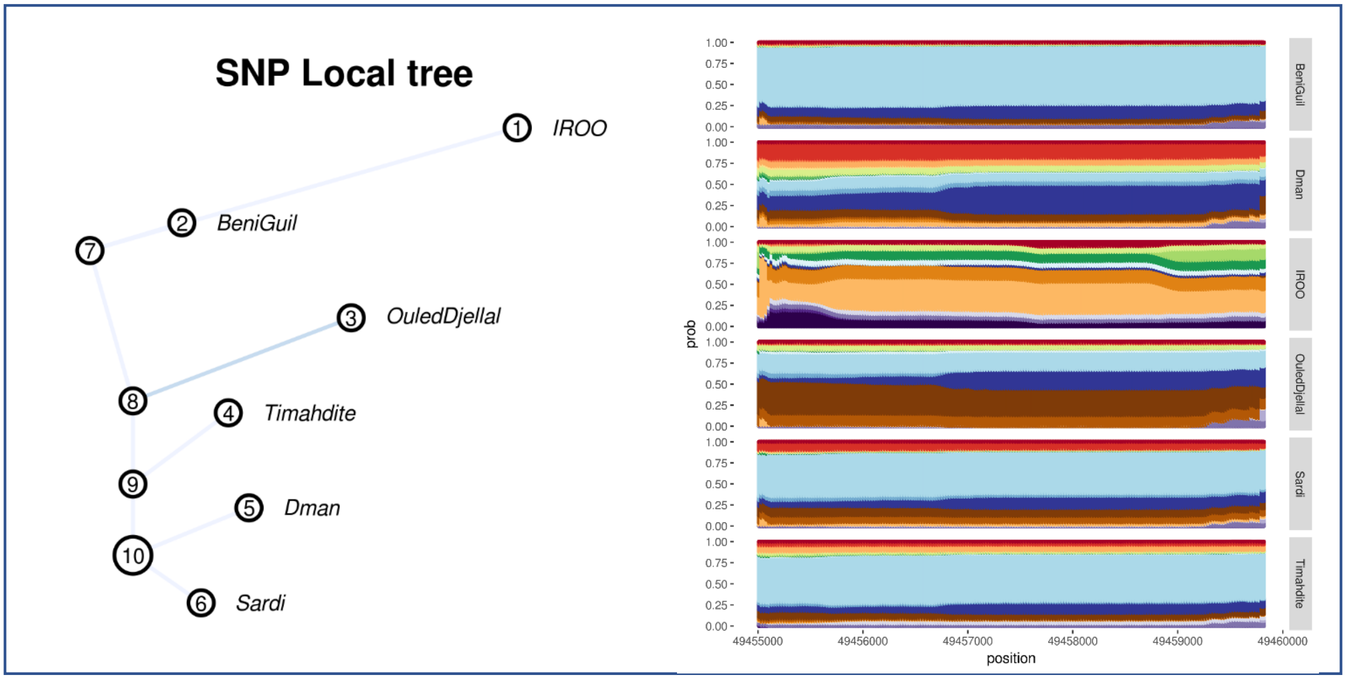

Figure S23: SNP local tree (left) and Haplotype cluster plot (right) of the region Chr17:49455690-49459271, showing the most differentiated breed: Ouled Jellal.

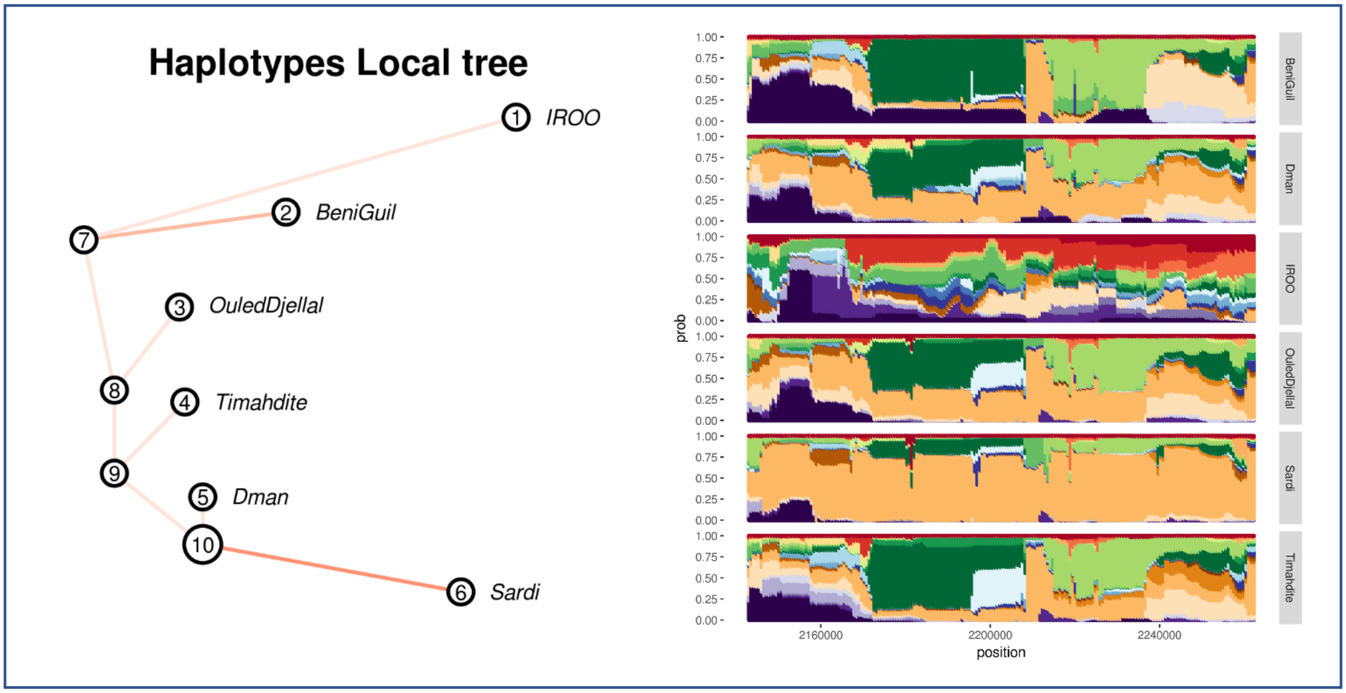

Figure S24: Haplotype local tree (left) and Haplotype cluster plot (right) of the region Chr19:2143797-2261064, showing the most differentiated breeds: BeniGuil and Sardi.

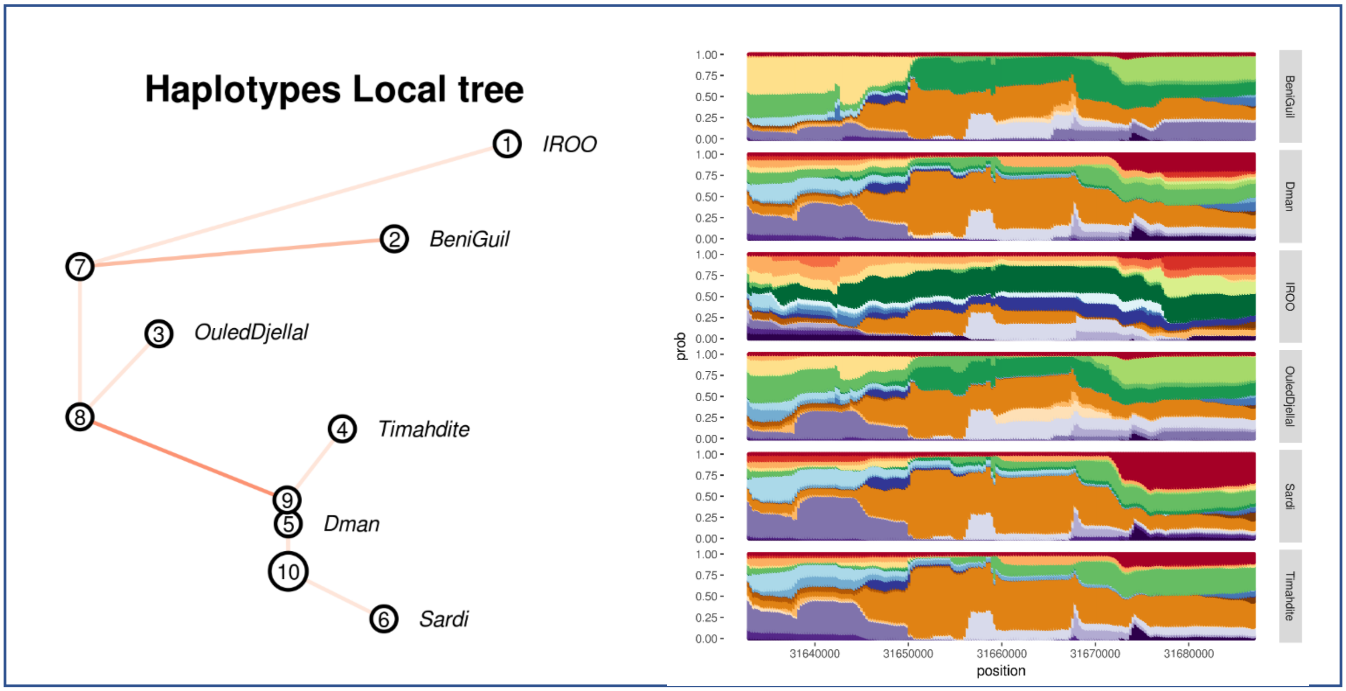

Figure S25: Haplotype local tree (left) and Haplotype cluster plot (right) of the region Chr19:31633503-31686458, showing the most differentiated breeds: BeniGuil, Timahdite, Dman and Sardi.

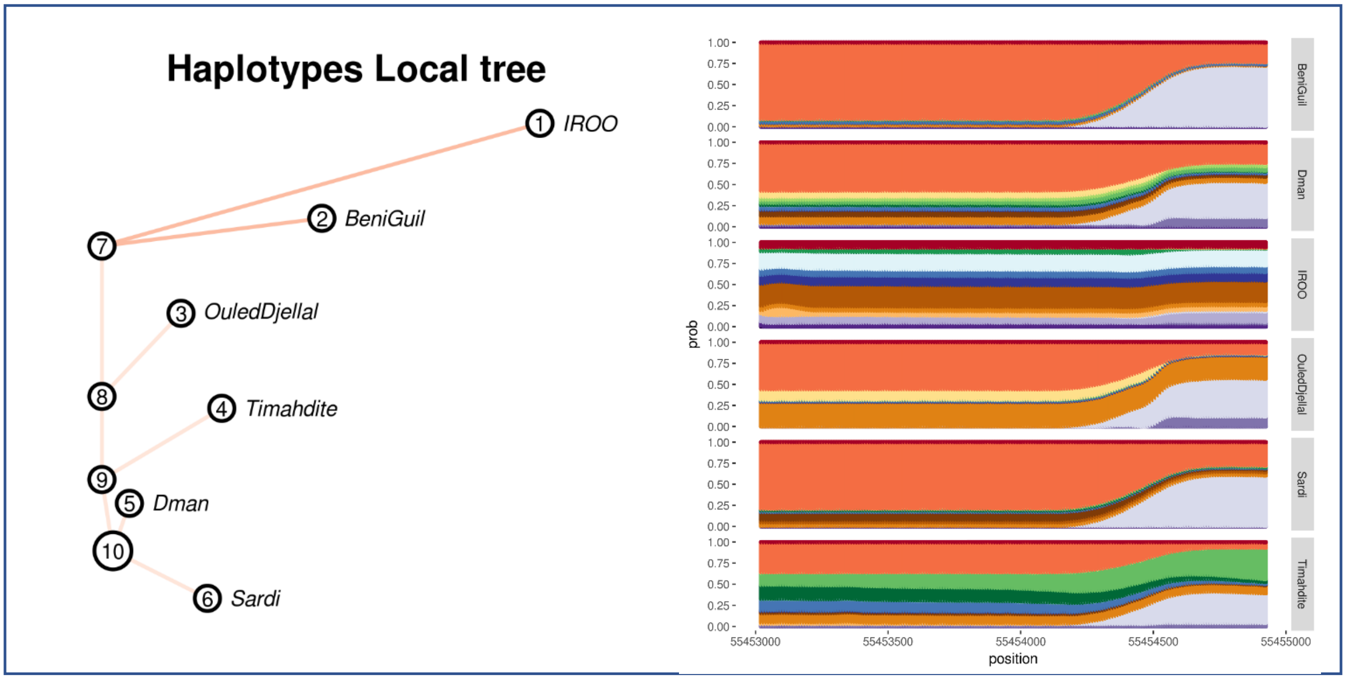

Figure S26: Haplotype local tree (left) and Haplotype cluster plot (right) of the region Chr19:55453233–55454316, showing the most differentiated breed: BeniGuil.

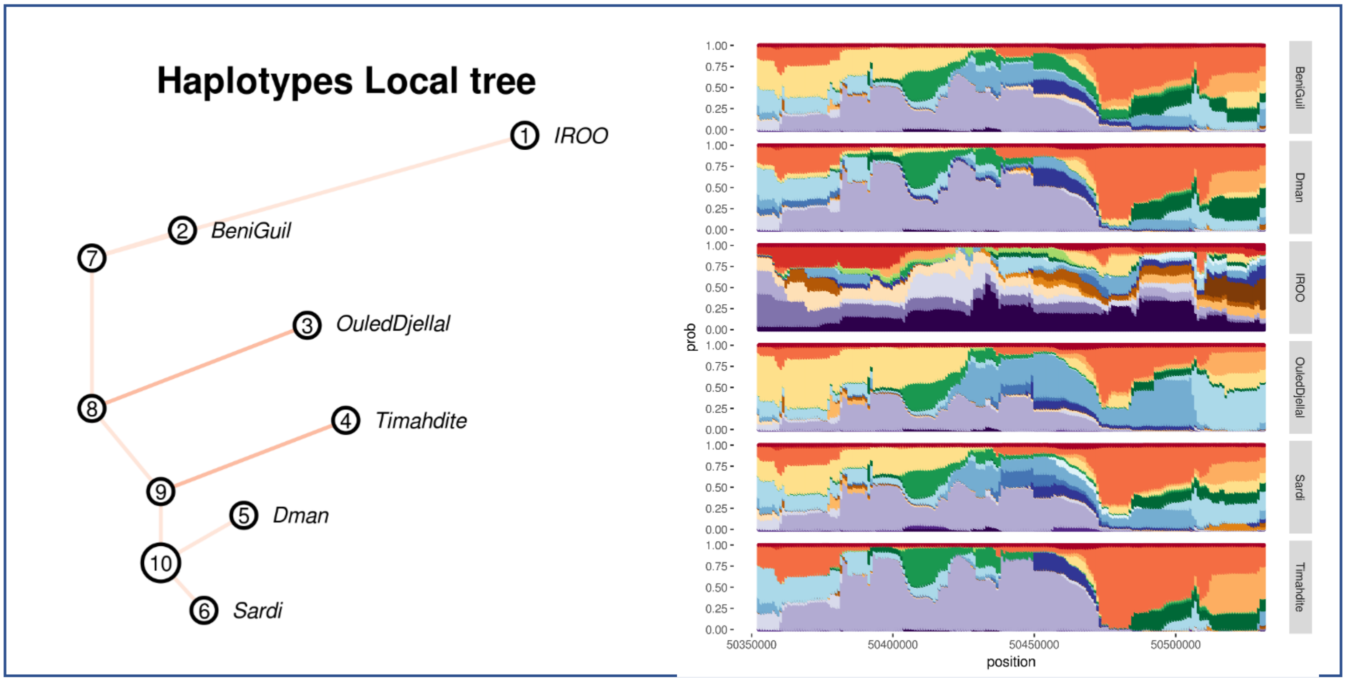

Figure S27: Haplotype local tree (left) and Haplotype cluster plot (right) of the region Chr20:50353200-50530655, showing the most differentiated breed: Timahdite and Ouled Jellal.

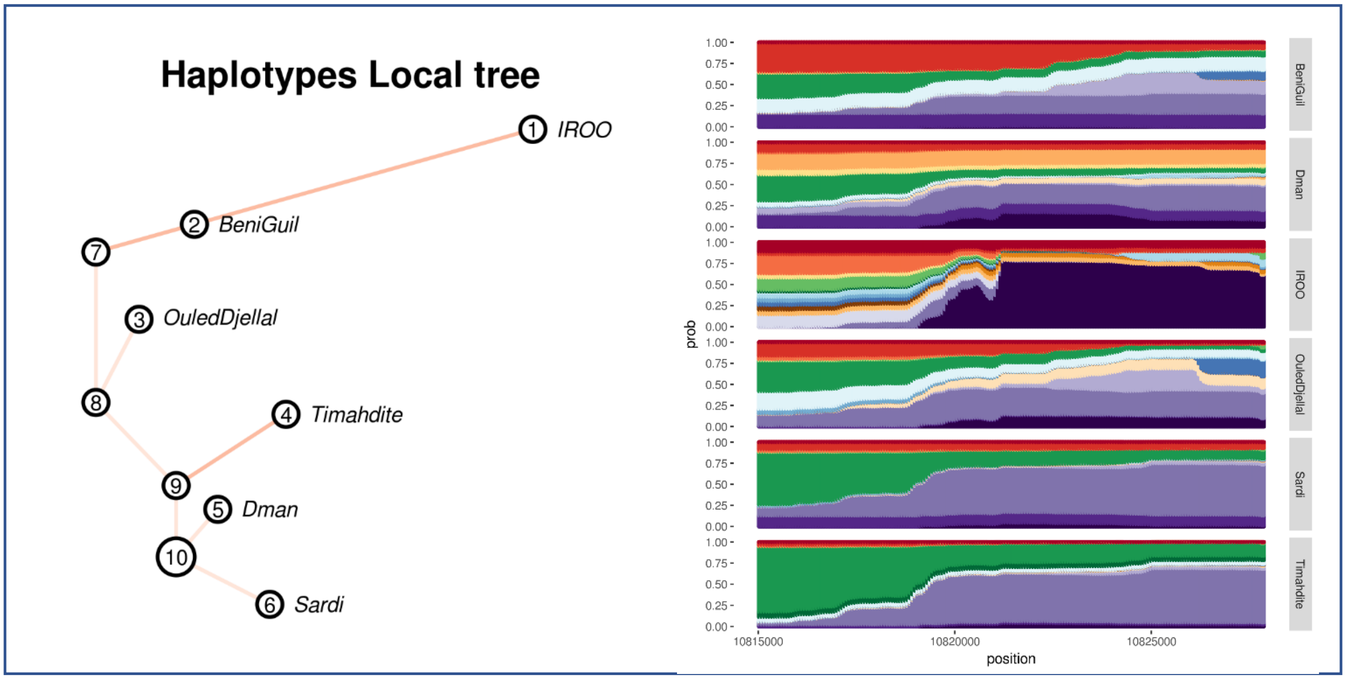

Figure S28: Haplotype local tree (left) and Haplotype cluster plot (right) of the region Chr21:10815838-10827827, showing the most differentiated breed: Timahdite.

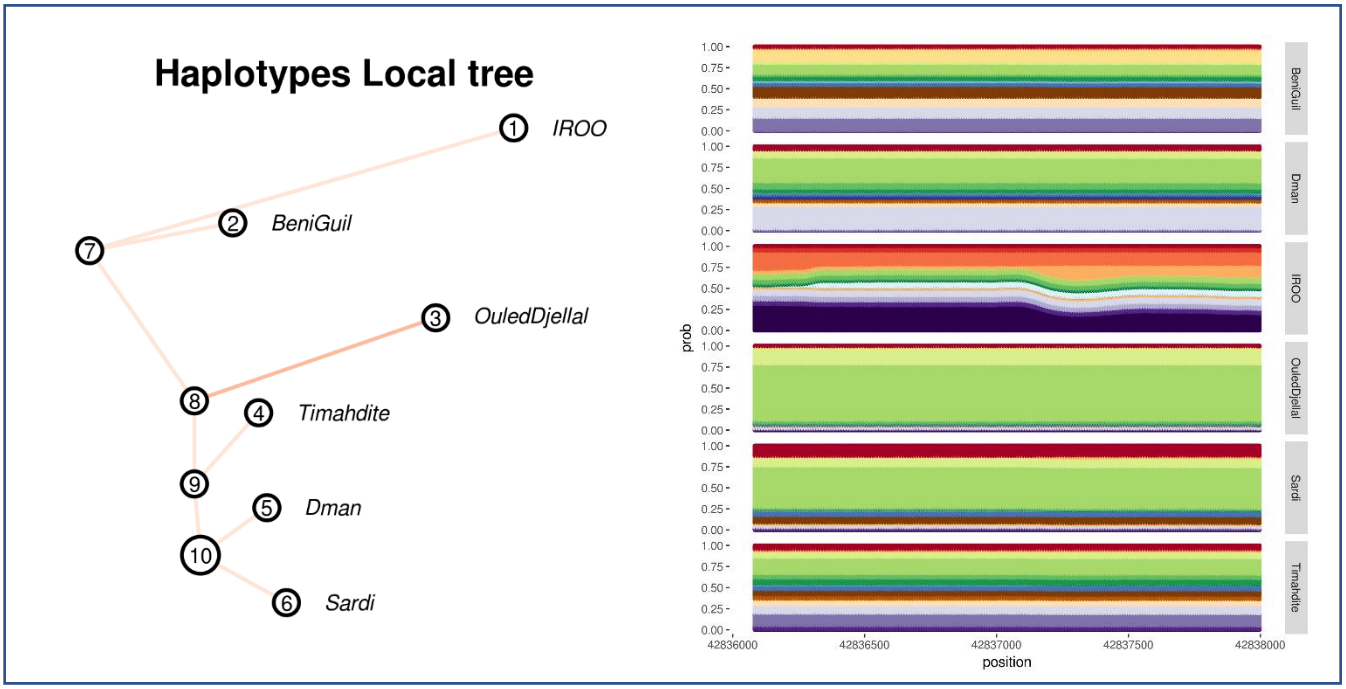

Figure S29: Haplotype local tree (left) and Haplotype cluster plot (right) of the region Chr21:42836462-42837960, showing the most differentiated breed: Ouled Jellal.

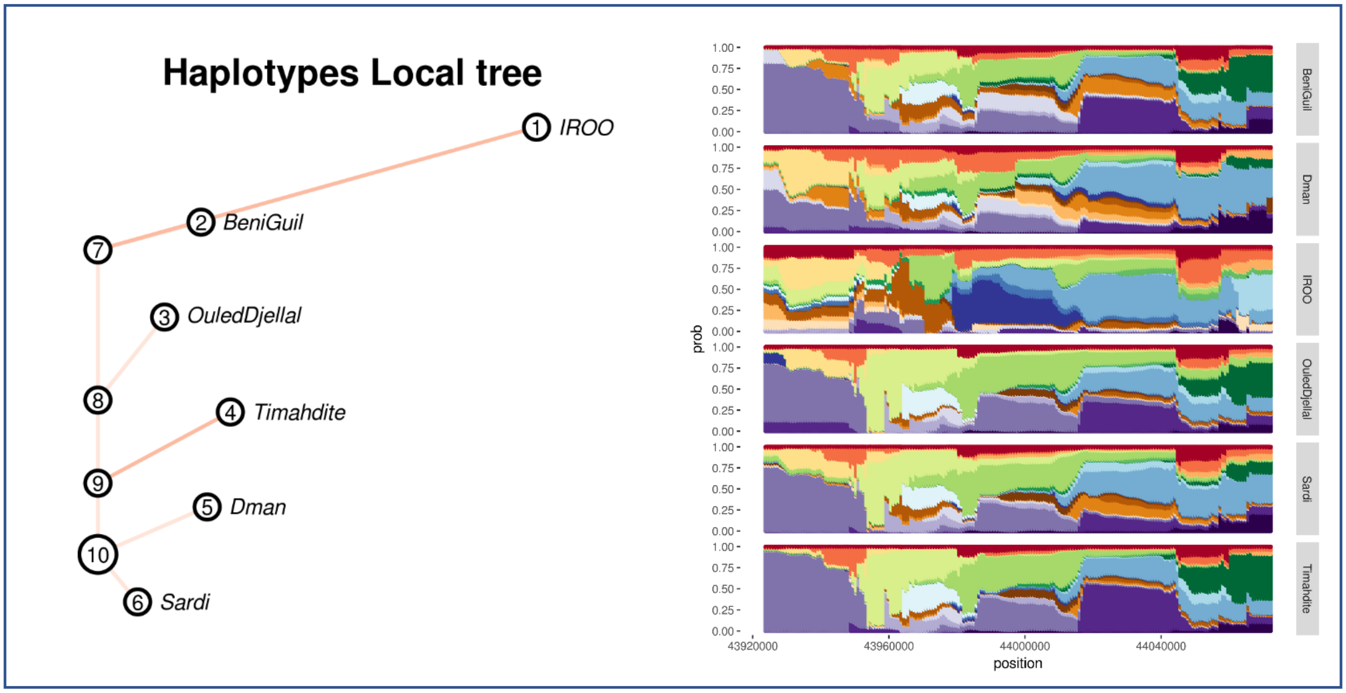

Figure S30: Haplotype local tree (left) and Haplotype cluster plot (right) of the region Chr23:43924165-44071386, showing the most differentiated breed: Timahdite and Dman.

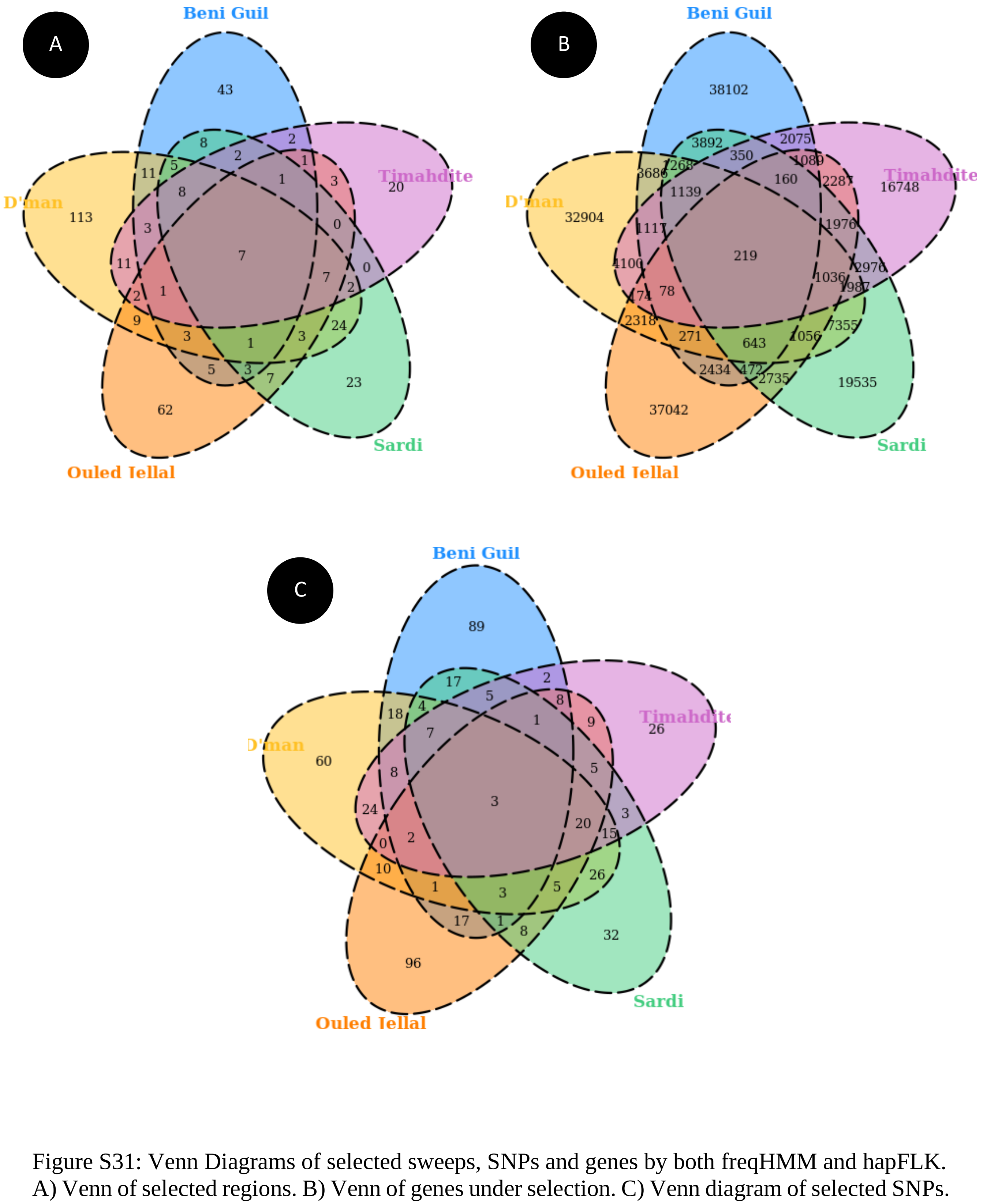

Figure S31: Venn Diagrams of selected sweeps, SNPs and genes by both freqHMM and hapFLK. A) Venn of selected regions. B) Venn of genes under selection. C) Venn diagram of selected SNPs.
