# Supplementary material for: Genomic Uniqueness of Local Sheep Breeds From Morocco
Source: Front Genet. 2021 Dec 2;12:723599. doi: 10.3389/fgene.2021.723599 (PMC8675355; doi:10.3389/fgene.2021.723599)
Supplement: Supplementary file 2 [file DataSheet2.zip › Table 1.DOCX]

Table S1: Demographic characteristics of the studied populations.

| Breed | Beni Guil | Dman | Ouled Jellal | Sardi | Timahdite | Wilds (from Asia) | Cosmopolitan breeds |
| --- | --- | --- | --- | --- | --- | --- | --- |
| Number of individuals | **6** | **30** | **8** | **27** | **16** | **13** | **22** |
| Mac | **1** | **2** | **1** | **2** | **1** | **1** | **1** |
| Mac_ld | **2** | **6** | **2** | **6** | **3** | **3** | **5** |
| Estimated r (recombination rate per bp per meiosis) | **8.7*10^-9^** | **9.96*10^-9^** | **9.98*10^-9^** | **9.90*10^-9^** | **9.82*10^-9^** | **9.6*10^-9^** | **9.87*10^-9^** |
| Estimated K parameter for freqHMM | **30** | **13** | **25** | **20** | **30** | **-** | **-** |
| Current effective population size (Ne) | **32399** | **29122** | **27718** | **28553** | **43654** | **1959** | **317** |
