# Supplementary material for: Genomic Uniqueness of Local Sheep Breeds From Morocco
Source: Front Genet. 2021 Dec 2;12:723599. doi: 10.3389/fgene.2021.723599 (PMC8675355; doi:10.3389/fgene.2021.723599)
Supplement: Supplementary file 2 [file DataSheet2.zip › Table 2.DOCX]

Table S2: Candidate regions identified by hapFLK in the corresponding selected breeds with the corresponding candidate genes and positions with the highest p-value (FDR<0.01%).

| Chrom | Position | Pos_Max | p-value | Breed undergoing selection | Candidate genes |
| --- | --- | --- | --- | --- | --- |
| 1 | 2729994 - 2746603 | 2730838 | 3.1e^-09^ | BeniGuil, Timahdite | TRAF3IP1 |
| 1 | 218627080 - 218627670 | 218627203 | 5.8e^-08^ | Dman | Intergenic |
| 1 | 230117531 – 230119919 | 230118700 | 1.3e^-08^ | Sardi, BeniGuil, OuledJellal | Intergenic |
| 1 | 265532246 – 265540828 | 265537811 | 5.3e^-08^ | Dman, Timahdite, BeniGuil | Intergenic |
| 2 | 1933721 – 1936398 | 1934636 | 2.32e^-08^ | Timahdite | ALLC |
| 2 | 84619111 – 84759276 | 84651917 | 3.38e^-13^ | Timahdite | BNC2, + intergenic |
| 2 | 128712616 - 128713554 | 128713202 | 1.51e^-07^ | Timahdite | Intergenic |
| 2 | 130242281 - 130247334 | 130244588 | 2.75e^-08^ | BeniGuil | TTN |
| 5 | 71731270 – 71732981 | 71731506 | 6.63e^-08^ | Sardi | Intergenic |
| 7 | 57083691 - 57108734 | 57085884 | 1.081e^-08^ | Ouled Jellal | Intergenic |
| 10 | 24458234 - 24459169 | 24458364 | 1.81e-^07^ | BeniGuil | Intergenic |
| 10 | 29363691 - 29806294 | 29447812 | 2.5e^-30^ | Dman | ENSOARG00000011616, RXFP2, + intergenic |
| 11 | 36801443 - 36805430 | 36803621 | 2.9e^-08^ | Dman | Intergenic |
| 13 | 29816181 - 29833006 | 29824188 | 3.95e^-11^ | OuledJellal | Intergenic |
| 13 | 46303939 - 46308888 | 46304996 | 4.27e^-08^ | Dman | RASSF2 |
| 13 | 56392318 - 56395466 | 56393699 | 2.7e^-09^ | Timahdite | EDN3 |
| 13 | 70139004 - 70139391 | 70139228 | 1.76e^-07^ | BeniGuil | Intergenic |
| 14 | 13329709 - 14250423 | 14199829 | 4.5e^-29^ | Sardi | VPS9D1, ZNF276, FANCA, SPIRE2, TCF25, MC1R, TUBB3, DEF8, + intergenic. |
| 16 | 34551942 - 34646856 | 34597927 | 9.4e^-17^ | Timahdite | Intergenic |
| 17 | 49455690 - 49459271 | 49457228 | 1.08e^-08^ | Ouled Jellal | Intergenic |
| 19 | 2143797 - 2261064 | 2185938 | 6.04e^-08^ | Sardi, BeniGuil | Intergenic |
| 19 | 31633503 - 31686458 | 31636613 | 2.6e^-08^ | BeniGuil, Timahdite, Dman, Sardi | MITF |
| 19 | 55453233 - 55454316 | 55454051 | 1.08e^-07^ | BeniGuil | Intergenic |
| 20 | 50353200 - 50530655 | 50514374 | 7.4e^-10^ | Timahdite, OuledJellal | GMDS, + Intergenic |
| 21 | 10815838 - 10827827 | 10825814 | 5.9e^-09^ | Timahdite | DLG2 |
| 21 | 42836462 - 42837960 | 42836741 | 6.51e^-08^ | Ouled Jellal | Intergenic |
| 23 | 43924165 - 44071386 | 44064928 | 2.3e^-09^ | Timahdite, Dman | Intergenic |
