# Supplementary material for: Genomic Uniqueness of Local Sheep Breeds From Morocco
Source: Front Genet. 2021 Dec 2;12:723599. doi: 10.3389/fgene.2021.723599 (PMC8675355; doi:10.3389/fgene.2021.723599)
Supplement: Supplementary file 2 [file DataSheet2.zip › Table 3.DOCX]

Table S3: Candidate regions identified, By FreqHMM, in the corresponding selected breeds.

| Chrom | Selected regions | Selected population |
| --- | --- | --- |
| 1 | 20364217 - 20532313 | BeniGuil |
| 1 | 37513816 - 37682776 | BeniGuil |
| 1 | 37733499 - 37864376 | BeniGuil |
| 1 | 63231754 - 63346482 | OuledJellal |
| 1 | 65913771 - 65919726 | Sardi |
| 1 | 79199243 - 79305560 | BeniGuil |
| 1 | 88629273 - 88671383 | OuledJellal |
| 1 | 107514771 - 107519665 | Dman |
| 1 | 129022747 - 129172254 | Timahdite |
| 1 | 131625732 - 131811871 | OuledJellal |
| 1 | 167371536 - 167415266 | BeniGuil |
| 1 | 172341434 - 172355013 | Dman |
| 1 | 185104275 - 185256938 | BeniGuil |
| 1 | 195595552 - 195748598 | Timahdite |
| 1 | 195604351 - 195742877 | Sardi |
| 1 | 203058908 - 203080618 | Dman |
| 1 | 209785808 - 209823413 | BeniGuil |
| 1 | 216135698 - 216148016 | Dman |
| 1 | 224354694 - 224368301 | Dman |
| 1 | 234317199 - 234382538 | Timahdite |
| 1 | 246841641 - 246877001 | OuledJellal |
| 1 | 246919205 - 246924827 | Dman |
| 1 | 259066625 - 259111082 | Sardi |
| 1 | 269946756 - 269959023 | OuledJellal |
| 1 | 65974821 - 65984779 | BeniGuil+Sardi |
| 1 | 71209698 - 71223688 | Dman+Sardi |
| 1 | 111196259 - 111218935 | Dman+Sardi |
| 1 | 119015206 - 119163925 | Dman+OuledJellal |
| 1 | 119376929 - 119419519 | OuledJellal+Sardi |
| 1 | 181482968 - 181549337 | OuledJellal+Sardi |
| 1 | 252160640 - 252168552 | Dman+OuledJellal |
| 1 | 266703750 - 266746549 | OuledJellal+Sardi |
| 1 | 237103029 - 237127153 | Dman+OuledJellal+Sardi |
| 1 | 199635778 - 199748894 | BeniGuil+Dman+Sardi+Timahdite |
| 2 | 346956 - 361983 | Dman |
| 2 | 3005309 - 3078866 | BeniGuil |
| 2 | 7795287 - 7912318 | Dman |
| 2 | 10787129 - 10836444 | OuledJellal |
| 2 | 15335383 - 15367350 | Dman |
| 2 | 16196552 - 16243181 | Dman |
| 2 | 20026120 - 20119318 | OuledJellal |
| 2 | 28881029 - 28886712 | Dman |
| 2 | 30308574 - 30320280 | Dman |
| 2 | 35699735 - 35941930 | BeniGuil |
| 2 | 41941580 - 41948843 | Dman |
| 2 | 46374178 - 46381138 | Dman |
| 2 | 51023821 - 51321269 | BeniGuil |
| 2 | 58727992 - 58741994 | Dman |
| 2 | 60787515 - 60835439 | OuledJellal |
| 2 | 63176861 - 63204165 | Dman+Sardi |
| 2 | 68061074 - 68131271 | BeniGuil+OuledJellal |
|  | 72192806 - 72214982 | BeniGuil+Dman+OuledJellal+Timahdite |
| 2 | 73653184 - 73669133 | Dman |
| 2 | 82056659 - 82167762 | BeniGuil+Sardi |
| 2 | 82621455 - 82678889 | BeniGuil |
| 2 | 86063863 - 86074817 | Dman |
| 2 | 98360371 - 98534208 | BeniGuil |
| 2 | 103947299 - 104049513 | BeniGuil+Sardi+Timahdite |
| 2 | 105750066 - 105777584 | OuledJellal |
| 2 | 106404417 - 106425810 | BeniGuil+Dman+Sardi |
| 2 | 106568458 - 106683216 | BeniGuil |
| 2 | 111362158 - 111611521 | BeniGuil+Dman |
| 2 | 113913807 - 114326027 | Sardi |
| 2 | 115171789 - 115317740 | Dman+OuledJellal+Sardi+Timahdite |
| 2 | 115918937 - 115980812 | OuledJellal+Sardi |
| 2 | 116439114 - 116459443 | Dman |
| 2 | 117946516 - 118061212 | BeniGuil+Dman |
| 2 | 122154666 - 122201720 | Timahdite |
| 2 | 122662005 - 122689130 | Dman+Sardi |
| 2 | 123185247 - 123215178 | Dman |
| 2 | 152171780 - 152330354 | OuledJellal |
| 2 | 182701813 - 182709691 | Dman |
| 2 | 184942926 - 185045571 | Dman+Sardi |
| 2 | 194863454 - 195006798 | OuledJellal |
| 2 | 198622022 - 198658355 | OuledJellal |
| 2 | 209562825 - 209668342 | OuledJellal |
| 2 | 213796810 - 213867903 | BeniGuil+Dman+OuledJellal |
| 2 | 214263411 - 214563016 | BeniGuil+Dman+Sardi+Timahdite |
| 2 | 218493122 - 218711661 | BeniGuil+Dman+Sardi+Timahdite |
| 2 | 219375021 - 219585555 | Dman+Sardi+Timahdite |
| 2 | 224584035 - 224635190 | Dman |
| 2 | 230152152 - 230225238 | BeniGuil |
| 2 | 232243484 - 232290999 | Dman |
| 2 | 234449925 - 234581855 | OuledJellal |
| 2 | 235615160 - 235666742 | Dman |
| 2 | 238293274 - 238301318 | Dman |
| 2 | 238855699 - 238871504 | Dman |
| 2 | 240794736 - 240809228 | Dman |
| 3 | 18260963 - 18357898 | Timahdite |
| 3 | 19667219 - 19719152 | Dman+Timahdite |
| 3 | 23550727 - 23607428 | OuledJellal |
| 3 | 32601412 - 32648627 | Sardi |
| 3 | 38068320 - 38111495 | Dman |
| 3 | 49845770 - 49870944 | Dman |
| 3 | 51653967 - 51754984 | OuledJellal |
| 3 | 57765176 - 57832078 | OuledJellal |
| 3 | 61983285 - 62000177 | Dman |
| 3 | 63393020 - 63493555 | BeniGuil+OuledJellal |
| 3 | 75391661 - 75402336 | Dman |
| 3 | 81058165 - 81113056 | Dman+OuledJellal |
| 3 | 87497178 - 87526047 | Dman+Sardi |
| 3 | 104881989 - 104904866 | Dman |
| 3 | 105028032 - 105074315 | OuledJellal |
| 3 | 107280055 - 107354904 | OuledJellal |
| 3 | 107842108 - 107927034 | BeniGuil |
| 3 | 111503031 - 111523669 | Dman+Timahdite |
| 3 | 112369332 - 112397214 | OuledJellal |
| 3 | 121258766 - 121288052 | Timahdite |
| 3 | 122503583 - 122533771 | Dman |
| 3 | 124802601 - 124966202 | Dman+Sardi |
| 3 | 124559412 - 124624482 | Dman |
| 3 | 129738227 - 129829205 | BeniGuil+OuledJellal+Sardi |
| 3 | 133594001 - 133655784 | Dman+OuledJellal+Sardi+Timahdite |
| 3 | 153763127 - 153879646 | BeniGuil+Dman+OuledJellal+Sardi+Timahdite |
| 3 | 155061548 - 155221514 | OuledJellal |
| 3 | 169980668 - 170017953 | Timahdite |
| 3 | 154327501 - 154394165 | Dman |
| 3 | 158293867 - 158304072 | Dman |
| 3 | 209658286 - 209721698 | OuledJellal+Sardi |
| 3 | 214472635 - 214515155 | Dman |
| 3 | 223994914 - 224026428 | Dman+Sardi |
| 4 | 1123411 - 1127253 | Dman |
| 4 | 1779224 - 1828106 | BeniGuil |
| 4 | 8405223 - 8423391 | Dman+Sardi |
| 4 | 23822177 - 23876109 | BeniGuil+Dman+OuledJellal+Sardi |
| 4 | 24446614 - 24465681 | Dman |
| 4 | 24446147 - 24487049 | OuledJellal |
| 4 | 35300451 - 35386867 | BeniGuil+Dman+Timahdite |
| 4 | 46053755 - 46120539 | OuledJellal |
| 4 | 48577921 - 48678247 | BeniGuil+Dman+Sardi+Timahdite |
| 4 | 51746377 - 51760096 | Dman |
| 4 | 61212240 - 61226878 | Sardi |
| 4 | 63039271 - 63124966 | BeniGuil+Dman+Sardi |
| 4 | 73261544 - 73318288 | Sardi |
| 4 | 85712184 - 85759609 | BeniGuil+Sardi |
| 4 | 87557368 - 87573777 | Dman |
| 4 | 91085209 - 91170246 | BeniGuil |
| 4 | 101280594 - 101305501 | Dman |
| 4 | 101335150 - 101382082 | Dman |
| 4 | 101689066 - 102074555 | BeniGuil+Dman+OuledJellal+Sardi+Timahdite |
| 4 | 102542918 - 102558139 | Dman+OuledJellal |
| 4 | 105053800 - 105067764 | Dman |
| 4 | 111795751 - 111902161 | BeniGuil |
| 4 | 118610284 - 118642940 | OuledJellal |
| 5 | 6339520 - 6348317 | Dman+Sardi |
| 5 | 6355470 - 6379867 | Dman |
| 5 | 52150797 - 52200247 | OuledJellal |
| 5 | 58035642 - 58103054 | Dman+OuledJellal |
| 5 | 59726500 - 59753244 | Dman |
| 5 | 74457498 - 74579533 | BeniGuil |
| 5 | 75027069 - 75045571 | BeniGuil+OuledJellal+Sardi |
| 5 | 78671145 - 78695042 | Dman |
| 5 | 91719097 - 91731163 | Dman |
| 5 | 94539287 - 94544620 | Dman |
| 5 | 99413999 - 99490760 | OuledJellal+Sardi |
| 5 | 100657735 - 100670093 | Dman |
| 5 | 101652017 - 101682492 | BeniGuil+Sardi |
| 5 | 104214995 - 104222140 | Dman |
| 5 | 106982269 - 107329817 | BeniGuil+Dman+OuledJellal+Sardi+Timahdite |
| 6 | 19992477 - 20038275 | OuledJellal+Timahdite |
| 6 | 24653786 - 24993512 | BeniGuil+OuledJellal+Timahdite |
| 6 | 32553604 - 32563681 | Dman |
| 6 | 33002214 - 33062570 | OuledJellal |
| 6 | 36284640 - 36339825 | Dman+OuledJellal+Sardi+Timahdite |
| 6 | 37626020 - 37639845 | Dman |
| 6 | 44752147 - 44923531 | Dman+OuledJellal+Sardi+Timahdite |
| 6 | 53584650 - 53619763 | Dman |
| 6 | 53635623 - 53812929 | OuledJellal |
| 6 | 69910301 - 70010991 | Sardi |
| 6 | 77052310 - 77063478 | Dman |
| 6 | 80199597 - 80212378 | BeniGuil+Dman |
| 6 | 108049487 - 108101458 | Timahdite |
| 6 | 116157907 - 116632256 | BeniGuil |
| 6 | 116446137 - 116705213 | Dman+Timahdite |
| 6 | 116843011 - 116979941 | Dman+Timahdite |
| 7 | 14716952 - 14791826 | Dman+Sardi+Timahdite |
| 7 | 27586647 - 27602808 | Dman |
| 7 | 43524035 - 43596080 | BeniGuil |
| 7 | 54103792 - 54229404 | OuledJellal |
| 7 | 55955341 - 56002274 | BeniGuil+Dman+Timahdite |
| 7 | 57101248 - 57456832 | OuledJellal |
| 7 | 71686024 - 71703076 | OuledJellal |
| 7 | 72517183 - 72548007 | Dman |
| 7 | 76978894 - 77024154 | Dman |
| 7 | 95857614 - 95862513 | Dman |
| 8 | 12013002 - 12039117 | Dman |
| 8 | 12569597 - 12638284 | BeniGuil+Dman+Sardi |
| 8 | 13976769 - 14038549 | OuledJellal |
| 8 | 16921673 - 16931227 | BeniGuil+Dman |
| 8 | 30410822 - 30416164 | Dman |
| 8 | 31381408 - 31513189 | BeniGuil+Sardi+Timahdite |
| 8 | 51150709 - 51258051 | Sardi |
| 8 | 52423032 - 52456568 | Timahdite |
| 8 | 56999623 - 57100945 | BeniGuil |
| 8 | 60456377 - 60468951 | Dman+Sardi |
| 8 | 61034954 - 61055290 | Dman |
| 8 | 75674286 - 75753583 | Timahdite |
| 8 | 76713198 - 76813669 | BeniGuil |
| 8 | 88012254 - 88042100 | Dman |
| 8 | 89646620 - 89671967 | BeniGuil+Dman+OuledJellal |
| 9 | 4773134 - 4806637 | OuledJellal |
| 9 | 5150269 - 5227250 | BeniGuil |
| 9 | 9785056 - 9831695 | Sardi |
| 9 | 23257413 - 23373442 | BeniGuil+Dman |
| 9 | 25170022 - 25189560 | Dman |
| 9 | 25443686 - 25541711 | Dman+Timahdite |
| 9 | 28484954 - 28517795 | Dman |
| 9 | 28525357 - 28537620 | Dman |
| 9 | 29766759 - 29782046 | Dman |
| 9 | 36515968 - 36617391 | BeniGuil |
| 9 | 49319885 - 49357910 | Dman+Sardi |
| 9 | 60398823 - 60459480 | OuledJellal |
| 9 | 61911214 - 62017638 | OuledJellal+Sardi |
| 9 | 65534022 - 65601905 | OuledJellal |
| 9 | 70199785 - 70225550 | Dman |
| 9 | 86636605 - 86867740 | BeniGuil |
| 10 | 7275557 - 7607570 | BeniGuil+OuledJellal+Sardi+Timahdite |
| 10 | 8054149 - 8400439 | BeniGuil |
| 10 | 8724620 - 8787999 | BeniGuil+Dman+Sardi |
| 10 | 18240209 - 18260207 | Dman |
| 10 | 19082840 - 19162258 | Dman+Sardi |
| 10 | 24553977 - 24635929 | BeniGuil+OuledJellal |
| 10 | 26206602 - 26350367 | Sardi |
| 10 | 32006295 - 32026769 | Dman |
| 10 | 36219490 - 36418784 | Timahdite |
| 10 | 36925017 - 36935408 | Dman |
| 10 | 54463676 - 54527533 | BeniGuil |
| 10 | 66031335 - 66087045 | OuledJellal |
| 11 | 10797595 - 10884166 | OuledJellal |
| 11 | 14396174 - 14415255 | Sardi |
| 11 | 18260984 - 18554847 | BeniGuil+Dman+Sardi+Timahdite |
| 11 | 24719717 - 24773705 | Dman+OuledJellal+Sardi |
| 11 | 38357402 - 38479200 | OuledJellal |
| 11 | 40311483 - 40343430 | Dman |
| 11 | 45421835 - 45434265 | Dman |
| 11 | 49146775 - 49196556 | BeniGuil |
| 11 | 51274662 - 51326707 | Sardi |
| 11 | 57879441 - 58075494 | BeniGuil |
| 11 | 59363306 - 59442466 | OuledJellal |
| 12 | 27794076 - 27814098 | Dman+Sardi |
| 12 | 38786233 - 38936200 | OuledJellal |
| 12 | 42741046 - 43021337 | Dman+Sardi |
| 12 | 49107272 - 49385302 | OuledJellal |
| 12 | 60628363 - 60632559 | Dman |
| 12 | 66266870 - 66288610 | Dman |
| 13 | 6500493 - 6598014 | OuledJellal |
| 13 | 17225339 - 17248599 | Dman+Sardi |
| 13 | 17571595 - 17614476 | BeniGuil |
| 13 | 26575511 - 26646581 | BeniGuil |
| 13 | 27226225 - 27396413 | OuledJellal |
| 13 | 29625052 - 29715754 | BeniGuil |
| 13 | 37412681 - 37421123 | Dman+Sardi |
| 13 | 48823392 - 49128402 | BeniGuil+Dman+Sardi+Timahdite |
| 13 | 49624329 - 49654140 | Dman+OuledJellal |
| 13 | 49670957 - 49789612 | Timahdite |
| 13 | 49808596 - 49992587 | Dman+OuledJellal+Sardi |
| 13 | 50137117 - 50636356 | BeniGuil+Dman+OuledJellal+Sardi+Timahdite |
| 13 | 56397872 - 56466572 | Dman |
| 13 | 62393490 - 62522047 | Timahdite |
| 14 | 7774232 - 7793482 | Dman |
| 14 | 34494403 - 34515079 | BeniGuil+Dman+OuledJellal |
| 14 | 48367809 - 48407031 | Dman |
| 15 | 1008841 - 1337208 | BeniGuil+Dman |
| 15 | 436496 - 536213 | OuledJellal |
| 15 | 3061137 - 3087236 | OuledJellal |
| 15 | 3247647 - 3251455 | Dman |
| 15 | 12030452 - 12079214 | OuledJellal |
| 15 | 13278652 - 13293241 | Dman |
| 15 | 21950846 - 22008215 | Dman+OuledJellal+Sardi+Timahdite |
| 15 | 42046064 - 42485157 | BeniGuil+Dman+OuledJellal+Sardi+Timahdite |
| 15 | 47507318 - 47735754 | BeniGuil+Dman+OuledJellal+Sardi+Timahdite |
| 15 | 48061517 - 48079031 | Dman+OuledJellal |
| 15 | 53466241 - 53504419 | BeniGuil+Dman |
| 15 | 64761349 - 64797782 | OuledJellal |
| 15 | 65567813 - 65580681 | Dman |
| 15 | 67970485 - 67985303 | BeniGuil+Dman |
| 16 | 19868632 - 19889361 | Dman |
| 16 | 19998047 - 20007346 | Dman |
| 16 | 21597732 - 21697328 | BeniGuil |
| 16 | 33294817 - 33305398 | Dman |
| 16 | 43235661 - 43281513 | Dman+Timahdite |
| 16 | 43682109 - 43706976 | BeniGuil+Dman |
| 16 | 49079826 - 49114725 | Timahdite |
| 16 | 51861610 - 51916601 | OuledJellal |
| 16 | 68018158 - 68035034 | Dman |
| 16 | 71231154 - 71326687 | Dman+Timahdite |
| 16 | 70639678 - 70987175 | BeniGuil+Sardi |
| 16 | 71340995 - 71466073 | OuledJellal |
| 16 | 71608687 - 71719576 | BeniGuil+Dman+Timahdite |
| 17 | 3841338 - 3884415 | Dman+Sardi |
| 17 | 9510420 - 9631499 | BeniGuil |
| 17 | 33058783 - 33173388 | OuledJellal |
| 17 | 34432047 - 34744005 | OuledJellal |
| 17 | 52351114 - 52441395 | Dman+OuledJellal+Sardi+Timahdite |
| 17 | 52763023 - 52873826 | OuledJellal+Timahdite |
| 17 | 59452834 - 59512304 | Dman |
| 17 | 62008649 - 62252402 | BeniGuil |
| 17 | 11938610 - 11947296 | Dman |
| 18 | 1158748 - 1267960 | BeniGuil+Sardi |
| 18 | 22629053 - 22808074 | Dman+OuledJellal |
| 18 | 23658266 - 23743937 | BeniGuil+Dman+Sardi |
| 18 | 32246388 - 32408044 | Dman+Sardi |
| 18 | 32638082 - 32653516 | Dman+Sardi |
| 18 | 45514036 - 45571251 | BeniGuil+Dman+Sardi+Timahdite |
| 18 | 45604833 - 45622008 | Dman |
| 18 | 48903395 - 48911607 | Dman |
| 18 | 66051806 - 66197995 | BeniGuil+Dman+OuledJellal+Sardi+Timahdite |
| 19 | 10036475 - 10057774 | Dman |
| 19 | 13936319 - 13969356 | OuledJellal |
| 19 | 20345328 - 20533954 | BeniGuil |
| 19 | 29626399 - 29774573 | BeniGuil |
| 19 | 43267934 - 43507813 | BeniGuil+OuledJellal |
| 19 | 59634907 - 59739278 | BeniGuil+Sardi |
| 19 | 60400894 - 60461422 | Sardi |
| 20 | 10851186 - 10868213 | Sardi |
| 20 | 15051939 - 15206917 | Dman+OuledJellal+Sardi+Timahdite |
| 20 | 18495431 - 18621501 | OuledJellal |
| 20 | 22254323 - 22284028 | Dman+Sardi |
| 20 | 39807889 - 39853141 | Dman+BeniGuil |
| 20 | 45755248 - 45843492 | OuledJellal |
| 20 | 49836117 - 49906986 | OuledJellal |
| 20 | 50106419 - 50340072 | Dman+Timahdite |
| 21 | 19986399 - 19996676 | Dman |
| 21 | 20289660 - 20301711 | Dman+OuledJellal+Timahdite |
| 21 | 39251120 - 39342751 | Dman+Timahdite |
| 21 | 39648580 - 39702625 | Sardi |
| 21 | 45951575 - 45970454 | Dman+OuledJellal+Timahdite |
| 21 | 46066212 - 46133866 | Sardi |
| 21 | 48336846 - 48372364 | Sardi |
| 22 | 7269322 - 7311181 | BeniGuil |
| 22 | 41147496 - 41226287 | Dman+Sardi |
| 23 | 14203301 - 14208993 | Dman |
| 23 | 36836294 - 36870569 | OuledJellal |
| 23 | 40563011 - 40655729 | Timahdite |
| 23 | 61045182 - 61116580 | OuledJellal |
| 23 | 61614422 - 61664245 | BeniGuil+OuledJellal |
| 24 | 13543324 - 13561163 | Dman+Sardi |
| 24 | 32724553 - 32758684 | Dman+Sardi |
| 24 | 32904926 - 32956396 | BeniGuil |
| 25 | 18887685 - 19013333 | OuledJellal |
| 25 | 2676142 - 2768712 | Dman |
| 25 | 28292311 - 28305958 | Dman |
| 25 | 34911158 - 34930814 | Dman |
| 26 | 38871884 - 38958368 | Dman+OuledJellal |
